# Compensatory evolution in NusG improves fitness of drug-resistant *M. tuberculosis*

**DOI:** 10.1038/s41586-024-07206-5

**Published:** 2024-03-20

**Authors:** Kathryn A. Eckartt, Madeleine Delbeau, Vanisha Munsamy-Govender, Michael A. DeJesus, Zachary A. Azadian, Abhijna K. Reddy, Joshua Chandanani, Nicholas C. Poulton, Stefany Quiñones-Garcia, Barbara Bosch, Robert Landick, Elizabeth A. Campbell, Jeremy M. Rock

**Affiliations:** 1 Laboratory of Host–Pathogen Biology, The Rockefeller University, New York, NY USA; 2 Laboratory of Molecular Biophysics, The Rockefeller University, New York, NY USA; 3 Department of Biochemistry, University of Wisconsin–Madison, Madison, WI USA; 4 Department of Bacteriology, University of Wisconsin–Madison, Madison, WI USA

**Keywords:** Pathogens, Bacterial genetics, Bacterial pathogenesis

## Abstract

Drug-resistant bacteria are emerging as a global threat, despite frequently being less fit than their drug-susceptible ancestors^1–8^. Here we sought to define the mechanisms that drive or buffer the fitness cost of rifampicin resistance (RifR) in the bacterial pathogen *Mycobacterium tuberculosis* (Mtb). Rifampicin inhibits RNA polymerase (RNAP) and is a cornerstone of modern short-course tuberculosis therapy^9,10^. However, RifR Mtb accounts for one-quarter of all deaths due to drug-resistant bacteria^11,12^. We took a comparative functional genomics approach to define processes that are differentially vulnerable to CRISPR interference (CRISPRi) inhibition in RifR Mtb. Among other hits, we found that the universally conserved transcription factor NusG is crucial for the fitness of RifR Mtb. In contrast to its role in *Escherichia coli*, Mtb NusG has an essential RNAP pro-pausing function mediated by distinct contacts with RNAP and the DNA^13^. We find this pro-pausing NusG–RNAP interface to be under positive selection in clinical RifR Mtb isolates. Mutations in the NusG–RNAP interface reduce pro-pausing activity and increase fitness of RifR Mtb. Collectively, these results define excessive RNAP pausing as a molecular mechanism that drives the fitness cost of RifR in Mtb, identify a new mechanism of compensation to overcome this cost, suggest rational approaches to exacerbate the fitness cost, and, more broadly, could inform new therapeutic approaches to develop drug combinations to slow the evolution of RifR in Mtb.

## Abstract (web summary)

In *Mycobacterium tuberculosis*, the fitness cost of rifampicin resistance is partially due to excessive RNA polymerase pausing and is rescued by mutations in the pro-pausing transcription factor NusG.

## Main

Antimicrobial resistance is a global threat, incurring both economic costs and loss of human lives. Although not generally discussed as part of the antimicrobial resistance crisis, drug-resistant Mtb accounts for one-quarter of all deaths due to antimicrobial resistance, and Mtb is the single leading cause of death due to infectious disease^11^. As is true for other bacterial pathogens^1–4^, drug resistance in Mtb is often associated with reduced fitness in the absence of drug pressure^5–8^. It was once thought that this reduced fitness might minimize patient-to-patient transmission and keep drug-resistant tuberculosis a localized problem, but this was not the case^14^. Mtb can acquire secondary ‘compensatory’ mutations that restore fitness and may promote more efficient pathogen transmission^15–18^.

**Fig. 1:**
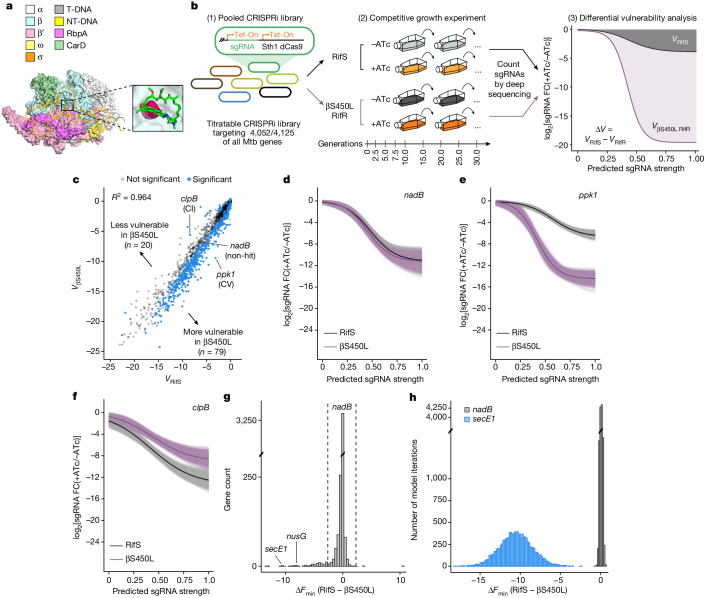
A genetic screen to identify differential vulnerabilities in βS450L RifR Mtb. **a**, Structural model of an Mtb RNAP transcription initiation complex bound to Rif. The sigma factor is shown as an orange outline. Inset, βSer450 is shown in pink. T-DNA, template DNA; NT-DNA, non-template DNA. **b**, Quantification of differential vulnerabilities (*V*). (1) An anhydrotetracycline (ATc)-inducible CRISPRi library was transformed into RifS and βS450L Mtb. Genes essential for in vitro growth^61^ were targeted with sgRNAs of varying predicted knockdown efficiencies. (2) Cultures were passaged and sgRNA abundance was assessed by deep sequencing at multiple timepoints. (3) A Bayesian model was then used to model the expression–fitness relationship (black and purple curved lines) for each gene. In brief, the *x* axis depicts predicted sgRNA strength (a proxy for the magnitude of target knockdown) and the *y* axis depicts bacterial fitness (predicted sgRNA log_2_-transformed fold change (log_2_FC) at 25 generations in the competitive growth experiment). The area above the expression–fitness curve was calculated to quantify gene vulnerability. Genes were called differentially vulnerable when the 95% credible region of the difference in gene vulnerabilities did not overlap 0. Δ*V* is differential vulnerability. Sth1 dCas9, *Streptococcus thermophilus* CRISPR1 dCas9. **c**, Scatter plot showing gene vulnerability (circles) in RifS and βS450L Mtb. Genes with significantly different vulnerabilities are shown in blue. CV, collateral vulnerability—that is, more vulnerable in βS450L; CI, collateral invulnerability—that is, less vulnerable in βS450L. **d**–**f**, Expression–fitness relationships for an example non-hit gene (*nadB*) (**d**), collateral vulnerability (*ppk1*) (**e**) and collateral invulnerability (*clpB*) (**f**). Light coloured lines represent the fits determined by 1,000 samples from the posterior distributions; dark lines represent the mean fits. **g**, Histogram showing the gene-level mean differences in the fitness cost imposed by the weakest possible sgRNAs (*F*_min_) between RifS and βS450L Mtb. Dashed lines mark two s.d. from the mean. **h**, Histograms showing the *F*_min_ distributions for *nadB* and *secE1* between RifS and βS450L Mtb.

One of the most potent first-line antibiotics to treat tuberculosis is rifampicin (Rif). Rif is the backbone of modern short-course tuberculosis treatment and its introduction effectively halved the duration of therapy^9^. Rif exerts its antibacterial effects by binding to the β-subunit of RNAP and blocking the extension of short RNA transcripts^10^ (Fig. 1a). Decades of Rif use have selected for RifR Mtb, which in 2021 caused up to 450,000 cases and 264,000 deaths worldwide^11^. Mtb develops resistance to Rif through mutations in the β-subunit that prevent Rif from binding, with the most frequent mutation being a single amino acid substitution, serine 450 to leucine (βS450L; Fig. 1a). βS450L accounts for up to 70% of all RifR Mtb in the clinic^12^. The βS450L mutation reduces Mtb fitness in the absence of Rif^5–7,12^, but Mtb can acquire compensatory mutations in the α, β or β′ subunits that restore fitness to levels equivalent to the drug-sensitive ancestor^15–17,19^.

RifR has pleiotropic effects on RNAP biochemistry and microbial physiology, but exactly how these differences reduce Mtb fitness are not fully understood. RifR mutations, including βS450L, alter the shape and chemical nature of the Rif-binding pocket. The Rif-binding pocket helps form the elongating RNA exit pathway^10^, and thus RifR mutations have the potential to affect the RNA elongation step of the transcription cycle^20^. Biochemical studies have shown that RifR mutations in RNAP can positively or negatively alter the stability of promoter open complexes, elongation speed or termination efficiency^20–22^. In Mtb, RifR decreases fitness and broadly impacts bacterial physiology. Several studies report alterations in the cell envelope in RifR strains^23^, including increased levels of phthiocerol dimycocerosates^24–26^, decreased levels of acylated sulfoglycolipids^25^, and changes in the levels of up to 2–6% of all lipid species^25^. RifR may also alter iron homeostasis, although this conclusion is muddled by conflicting reports about whether levels of mycobactin—the iron-scavenging siderophore—are higher^27^ or lower^25^ in RifR Mtb, and the lack of such an iron-response signature across diverse RifR clinical isolates^27^.

Here we sought to define the mechanisms driving or buffering the fitness cost of βS450L Mtb. Given the often subtle and genetic background-dependent effects of RifR on the Mtb transcriptome and proteome^27^, we chose to use a functional genomics approach. Comparative genome-scale CRISPRi screening in RifS and βS450L Mtb identified approximately 150 genes involved in diverse cellular processes that are more or less sensitive to genetic silencing in βS450L, which we refer to as differential vulnerabilities. Among these hit genes, we found that the universally conserved, essential transcription factor *nusG* is crucial for the fitness of βS450L Mtb. Combining this approach with a bacterial genome-wide association study, we further found that *nusG* is under positive selection in RifR clinical Mtb isolates. We show that clinically observed NusG mutants reduce the pro-pausing and termination activity of NusG and compensate for the increased pausing and termination properties of βS450L RNAP. NusG mutants thereby increase the fitness of βS450L Mtb. Collectively, these results define excessive RNAP pausing and termination as molecular mechanisms that drive the fitness cost of βS450L, identify a new mechanism of compensation to overcome this cost, suggest rational approaches to exacerbate the fitness cost, and, more broadly, could inform new therapeutic approaches to develop drug combinations to slow the evolution of RifR in Mtb.

## Identifying differential vulnerabilities

To identify differential vulnerabilities in RifR βS450L (hereafter βS450L) Mtb, we used our tunable Mtb CRISPRi library^28,29^ (Fig. 1b). This library consists of 96,700 unique single guide RNAs (sgRNAs) that target around 98% of all annotated Mtb genes. Knockdown tuning is achieved by varying the targeted protospacer adjacent motif (PAM), varying the length of the sgRNA targeting sequence, or both. Biosafety concerns dictated that we perform these screens in an isogenic pair of RifS or βS450L Mtb biotin auxotrophs. The biotin auxotroph (Δ*bioA*) grows similarly to prototrophic Mtb in the presence of 2 μM biotin but cannot establish infection in mice. We confirmed that βS450L Δ*bioA* had a growth defect relative to RifS Δ*bioA* Mtb (Supplementary Fig. 1a). All validation and follow-up experiments were conducted in the prototrophic parental H37Rv RifS or βS450L strains.

Following transformation of the CRISPRi library into RifS and βS450L Δ*bioA* Mtb, we carried out two separate competitive growth experiments. Triplicate cultures for each library were passaged for approximately 30 generations in the presence or absence of the CRISPRi inducer ATc (Fig. 1b). Every 2.5 or 5 generations, we collected genomic DNA, analysed sgRNA abundance by deep sequencing, and calculated the log_2_-transformed fold change of sgRNA read counts with or without ATc. Growth phenotypes were well correlated among triplicate screens (Supplementary Fig. 1b–e). Whole-genome sequencing of the final competitive growth timepoint confirmed that compensated βS450L mutants did not take over the culture at late timepoints. sgRNA depletion data were then used to calculate gene vulnerability using a multilevel Bayesian model, similar to that described previously^28^. As opposed to binary gene essentiality calls, the vulnerability model generates expression–fitness relationships for every targeted gene by relating the predicted magnitude of target knockdown (as inferred by the sgRNA strength) to the resulting fitness cost imposed on the bacteria. The model is iterated 12,000 times to generate distributions of vulnerability values for each gene. Differential vulnerabilities are defined as those genes for which the 95% credible region of differences in gene vulnerability do not overlap 0.

As expected, most genes were similarly vulnerable in the RifS and βS450L strains (*R*^2^ = 0.964; Fig. 1c and Supplementary Table 1). For example, the nicotinamide adenine dinucleotide (NAD) biosynthetic enzyme *nadB* showed very similar expression–fitness relationships between the two Mtb strains (Fig. 1d). However, 99 genes displayed a statistically significant difference in vulnerability (Fig. 1c). Most differentially vulnerable genes were more sensitive to knockdown in βS450L (*n* = 79 genes; Fig. 1c,e), but some were less sensitive (*n* = 20 genes; Fig. 1c,f). Drawing parallels with collateral interactions between drugs and resistance mutations^30^, we refer to these differentially vulnerable genes as collateral vulnerabilities or collateral invulnerabilities, respectively.

We observed that a subset of genes did not exhibit a dose–response relationship between sgRNA strength and bacterial fitness, and the standard vulnerability model did not accurately capture the expression–fitness relationship for these genes (Supplementary Fig. 1f). These genes tended to be those that were already very vulnerable in the RifS strain and became even more vulnerable in βS450L. To capture these hit genes, we identified the fitness cost imposed by the weakest possible sgRNAs (defining the fitness minimum (*F*_min_)), or in effect where the expression–fitness relationship crossed the *y* axis (Supplementary Fig. 1g,h). This approach identified 73 hit genes (Fig. 1g,h and Supplementary Table 1), including 53 genes that were not identified in the original model.

## Translation is a collateral vulnerability in βS450L

**Fig. 2:**
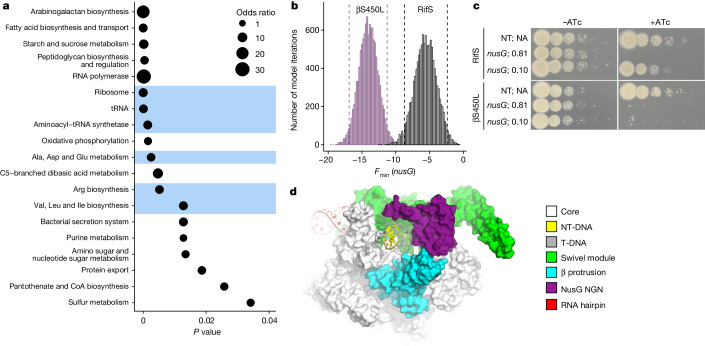
Pathway analysis identifies differentially vulnerable processes in βS450L Mtb. **a**, Bubble plot of enriched (*P* ≤ 0.05) pathways for the genes identified as differential vulnerabilities in βS450L. Pathways highlighted in blue are related to translation. *P* values and odds ratios were determined by Fischer’s exact test. **b**, The distribution of *nusG* *F*_min_ values in RifS (grey) and βS450L (purple) Mtb. Dashed lines represent the 95% credible region. **c**, Phenotypic consequences of strong and hypomorphic knockdown of *nusG* (sgRNA predicted strength = 0.81 and 0.10, respectively, where 1.0 represents the strongest possible sgRNA) in RifS and βS450L Mtb. NT, non-targeting sgRNA. **d**, Structure of paused Mtb RNAP bound to NusG. The RNAP swivel module was defined by Delbeau et al.^13^.

We next performed functional enrichment analysis to identify pathways that are more vulnerable to inhibition in βS450L Mtb (Fig. 2a). This approach identified genes involved in peptidoglycan, arabinogalactan and mycolic acid biosynthesis as more sensitive to CRISPRi inhibition in βS450L, consistent with the known cell envelope differences between RifS and RifR Mtb^23^. Many metabolic processes were also enriched as more vulnerable in βS450L, potentially consistent with prior observations of altered metabolism in RifR Mtb^27^. Of note, many genes that are critical for translation were also identified as more vulnerable in βS450L, including aminoacyl-tRNA synthetases, tRNAs, ribosomal proteins, translation initiation and elongation factors, and several amino acid biosynthetic pathways (Fig. 2a).

We next explored the reasons why translation might be more sensitive to CRISPRi inhibition in βS450L. Biochemical studies show that βS450L RNAP elongates more slowly and terminates more frequently than RifS RNAP in vitro^20^. It is also known that during the process of transcription–translation coupling (TTC), the translating ribosome can stimulate transcription elongation by RNAP either directly^31^ or mediated by the universally conserved transcription factor NusG^32,33^. Although TTC has yet to be directly demonstrated in Mtb, recent evidence suggests that, similar to *E. coli*, transcription and translation are coupled in Mtb^34^. Thus, we hypothesized that translation may promote fitness in βS450L by maintaining coupling of the ribosome to a slow, pause-prone RNAP to promote more efficient transcription elongation. *nusG* is a strong collateral vulnerability in βS450L (Figs. 1g and 2b,c), potentially consistent with the hypothesis that TTC may be important to promote βS450L fitness.

Mtb NusG is composed of two domains, a NusG N-terminal (NGN) domain and a C-terminal Kyprides, Ouzounis, Woese (KOW) domain. The NGN domain interacts with RNAP^13^ and the KOW domain interacts potentially with several proteins, including the S10 ribosomal protein^35^. By interacting with the ribosome, NusG could serve as a bridge mediating TTC, at least as demonstrated in *E. coli*^32,33,36^. But the NGN domain also modulates transcription pausing via distinct contacts with RNAP and the DNA (Fig. 2d). In bacteria, pause-inducing sequences in the DNA and RNA reversibly halt RNAP elongation, thereby favouring RNAP kinetics towards termination^37^. RNAP pausing and termination are additionally aided by *trans*-acting factors such as NusG. Mtb NusG promotes pausing and termination by stabilizing a swivelled RNAP conformation, which slows nucleotide addition and thus RNA elongation^13,38,39^. This pro-pausing activity of Mtb NusG is mediated via unique interactions between the NusG NGN domain, the RNAP β-protrusion domain, and the NT-DNA, which stabilize the paused, swivelled RNAP state^13^.

*nusG* is a strong collateral vulnerability, and thus the presence of NusG promotes βS450L fitness. The positive effect of NusG on βS450L fitness may be explained by its anti-pausing role in mediating TTC, although other potential explanations exist. By contrast, the pro-pausing and termination activity of the NusG NGN domain might be expected to be detrimental to βS450L fitness. Thus, we explored whether the pro-pausing and termination activity of NusG might in fact be a source of the fitness cost in βS450L and, if so, whether it might be under selective pressure to mutate to restore fitness in βS450L Mtb.

## NusG is diversifying in RifR Mtb

**Fig. 3:**
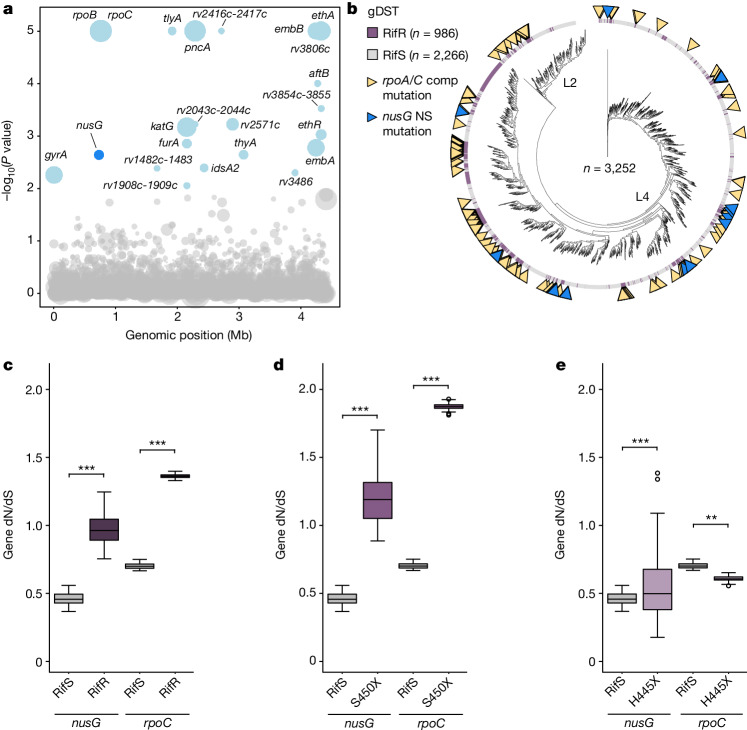
NusG mutations are associated with RifR in clinical Mtb isolates. **a**, Manhattan plot showing the genetic association with RifR among 3,252 clinical Mtb isolates from Peru. Each circle represents a gene or intergenic region in the Mtb genome. The *y* axis represents uncorrected phyOverlap *P* values (blue, *P* ≤ 0.05). Circle sizes are scaled by the number of independent mutations observed for that gene or intergenic region. *P* values were derived from 50,000 permutations of mutations events, as described in Methods. **b**, Phylogenetic tree of Peruvian Mtb isolates from **a**. *Mycobacterium canetti* was included as an outgroup; isolates from lineages 2 and 4 (L2 and L4) are indicated. Purple lines represent isolates identified as RifR by genotypic drug susceptibility testing (gDST). Yellow triangles mark isolates with a known compensatory (comp) mutation in *rpoA/C* (Supplementary Table 2) and blue triangles mark isolates harbouring a nonsynonymous (NS) mutation in *nusG*. **c**–**e**, Box plots showing the ratio of nonsynonymous to synonymous mutations (dN/dS) for *nusG* and *rpoC* in genotypically predicted RifS (*nusG*, *n* = 1,365; *rpoC*, *n* = 15,834) and RifR (*nusG*, *n* = 350; *rpoC*, *n* = 10,418) Mtb (**c**), restricting the analysis to only RifR strains with mutations in Ser450 (*nusG*, *n* = 270; *rpoC*, *n* = 7,898) (**d**), or restricting the analysis to only RifR strains with mutations in His445 (*nusG*, *n* = 26; *rpoC*, *n* = 996) (**e**). X indicates any amino acid except Ser (S450) or His (H445). The centre line represents the mean, box edges delineate top and bottom quartiles, and whiskers indicate the minimum and maximum dN/dS values. *P* values by independent two-sample *t*-test. Two-sided *P* values: **P = 1.14 × 10^−3^, ***P ≤ 0.0001.

To answer this question, we sought to determine whether *nusG* is under positive selection in clinical Mtb isolates. Using a cohort of Mtb clinical isolates from Peru^40^, we first performed a bacterial genome-wide association study to identify genetic variants associated with genotypically predicted RifR. This approach identified several well-known genes involved in Mtb drug resistance including *rpoB*, *pncA* and *embB*, as well as variants in the β′ subunit (*rpoC*) that are known to compensate for the fitness cost of RifR^15,16^ (Fig. 3a,b and Supplementary Tables 2 and 5). The association of RifR with resistance variants to other first and second-line drugs reflects the multiple resistance mutations present in many multidrug-resistant (MDR) Mtb isolates. Potentially consistent with our hypothesis, variants in *nusG* were also statistically associated with RifR in this cohort (Fig. 3a,b and Supplementary Fig. 2a). Although this association did not meet the significance cut-off when applying a genome-wide multiple test correction, *nusG* variants were nevertheless associated with RifR with a *P* value similar to that of well-known resistance-associated genes such as *gyrA*, *katG* and others, suggesting that *nusG* variants are indeed enriched in RifR Mtb.

To investigate this phenomenon further, we leveraged an in-house database of around 50,000 publicly available whole-genome sequences from Mtb clinical isolates from around the world^41^. Analysis of the ratio of nonsynonymous to synonymous mutations (dN/dS) showed *nusG* to be under purifying selection in RifS Mtb (Fig. 3c), consistent with the fact that *nusG* is an essential gene^13^. By contrast, *nusG* was under stronger diversifying selection in RifR Mtb, as evidenced by a higher dN/dS ratio in RifR as compared to RifS Mtb. Closer analysis revealed that *nusG* selection was specific to certain RifR *rpoB* alleles (Supplementary Fig. 2b,c and Supplementary Tables 2 and 3). For example, *nusG* is under comparatively diversifying selection in βS450X (where X indicates any amino acid except Ser) but under purifying selection in βH445X (Fig. 3d,e), a pattern very similar to that observed for variants in *rpoC*^16,17^ (Fig. 3c–e). This phenomenon could be explained by the fact that some RifR RNAP mutants such as βS450L are slow and over-pause or over-terminate, whereas βH445Y RNAPs are fast and less likely to pause or terminate^12,20,22^. We would expect selective pressure to reduce the pro-pausing activity of NusG to be specific to slow, pause-prone RifR RNAP mutants. *nusG* variants are not commonly found in RifR strains with known compensatory mutations in *rpoA*, *rpoB* or *rpoC* (Supplementary Fig. 2d and Supplementary Tables 2 and 3). Collectively, these results show that *nusG* is evolving convergently and may represent a new class of compensatory mutations in RifR Mtb.

## Mutations reduce NusG pro-pausing activity

**Fig. 4:**
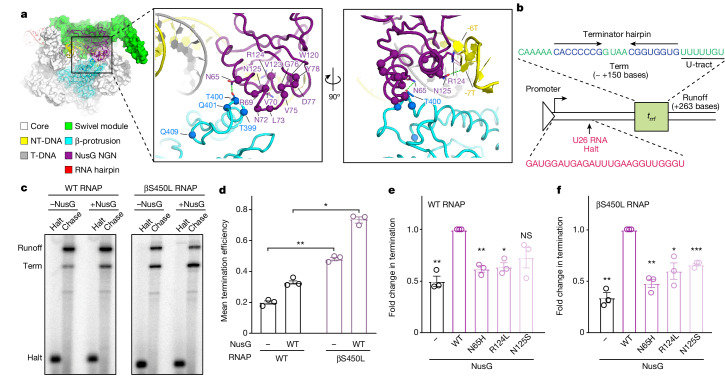
Clinical strain mutations decrease the pro-pausing activity of NusG. **a**, Cryo-EM structure of a NusG-bound paused elongation complex from Mtb. The boxed area shows the location of mutations observed in RifR clinical Mtb isolates. Interactions between NusG and the β-protrusion or non-template DNA are shown in green dashed lines^13^. **b**, Promoter template with the Mtb *rrf* termination sequence for in vitro transcription assays. The experimental schematic was followed for data shown in **c**–**f**. **c**, In vitro termination by Mtb RNAP on a promoter-initiated template with or without wild-type (WT) NusG. The U26, C-less halt site is labelled (Halt), as well as approximately +150 base termination site (Term) and +263 base runoff (Runoff). **d**, Termination efficiencies of wild-type and βS450L RNAP in the presence (two-sided adjusted *P* value = 0.039) or absence (two-sided adjusted *P* value = 0.002) of wild-type NusG. Data are mean ± s.d. from three experimental replicates. **e**, Fold change in termination (∆*T*) relative to wild-type RNAP plus wild-type NusG. Data are mean ± s.d. from three experimental replicates. ∆*T* for each NusG protein was calculated from kinetics of the elongation versus termination process as described by von Hippel and Yager^62,63^ based on the relationships between termination efficiency and the rates and activation energies of termination (Methods). Left to right, two-sided adjusted *P* value: 0.009, 0.006, 0.024 and 0.057. **f**, Fold change in termination as in **e**, relative to βS450L RNAP plus wild-type NusG. Left to right, two-sided adjusted *P* value: 0.009, 0.006, 0.038 and 8.7 × 10^−4^. (**d**–**f**) Independent two-sample *t*-test, with multiple hypothesis correction by Benjamini, Krieger and Yekutieli method. *P ≤ 0.05, **P ≤ 0.01, ***P ≤ 0.001; NS, not significant.

Homoplastic *nusG* mutations occurred exclusively in the NGN domain (Supplementary Table 2). To predict the functional consequences of these mutations, we mapped them onto the Mtb NusG structure^13^. Notably, these mutations clustered at the unique, pro-pausing interfaces between NusG and either the β-protrusion or the NT-DNA^13^ (Fig. 4a). These mutations would be expected to weaken the interaction between the NusG NGN domain and the β-protrusion or the NT-DNA and thereby weaken the pro-pausing and termination activity of NusG. Consistent with this interpretation, we also observed homoplastic mutations in the β-protrusion that are found exclusively in RifR Mtb (Fig. 4a and Supplementary Table 2). Similar to NusG NGN mutations, these β-protrusion mutations are anticipated to reduce the strength of the NusG–β-protrusion interaction. Notably, none of these mutations would be predicted to prevent NusG from interacting with RNAP because of the extensive NusG–RNAP contacts via the swivel module^13^. Thus, these *nusG* and β-protrusion mutants would be expected to specifically weaken pro-pausing and termination activity but maintain other functions of the NusG–RNAP interaction.

To test the prediction that the homoplastic NusG mutants weaken its pro-pausing activity, we purified recombinant Mtb wild-type RNAP, βS450L RNAP, wild-type NusG, and three of the most common NusG variants observed in clinical RifR Mtb isolates. We then assessed the effect of each variant on termination (and, by inference, RNAP pausing) using in vitro termination assays (Fig. 4b). In the absence of NusG, βS450L RNAP displayed increased termination compared to RifS RNAP^20^ (Fig. 4c,d). In the presence of NusG, both RifS and βS450L RNAPs displayed increased levels of termination^13^ (Fig. 4c,d). Consistent with the hypothesis that the clinical NusG variants weaken the interaction between NusG NGN and the β-protrusion and thereby decrease RNAP pausing and termination^13^, all three NusG mutants showed reduced levels of pro-pausing and termination activity when paired with either wild-type or βS450L RNAP (Fig. 4e,f). We note that NusG variants do not reduce βS450L RNAP termination to wild-type RNAP plus wild-type NusG levels in this assay, suggesting that RNAP pausing and termination levels may remain partially elevated in vivo. All NusG variants bind to RNAP similarly to wild-type NusG (Supplementary Fig. 2e,f). These data suggest that NusG and β-protrusion variants reduce RNAP swivelling dwell time and consequently decrease pausing and hyper-termination, rescuing the fitness defect in βS450L Mtb.

## NusG mutations boost βS450L fitness

**Fig. 5:**
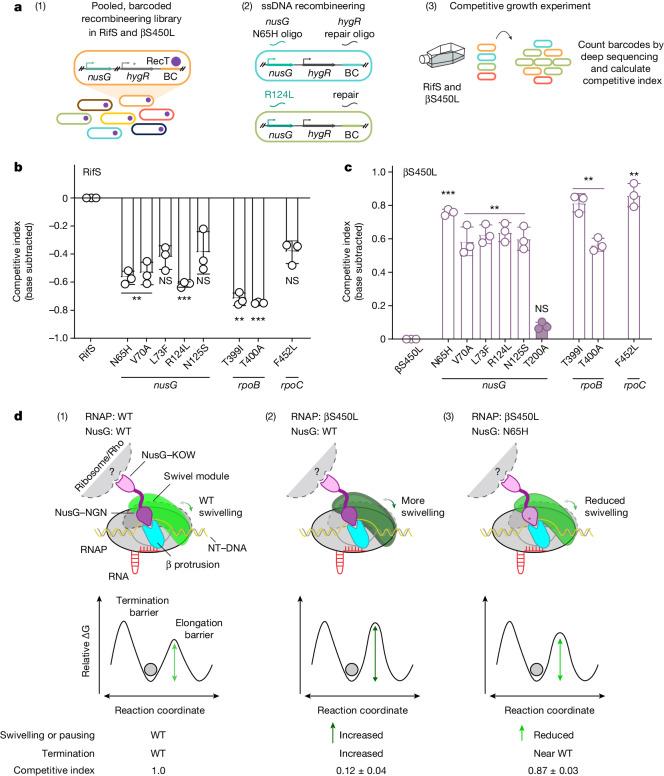
Compensatory mutations in *nusG* increase the fitness of βS450L Mtb. **a**, Experimental design to quantify the competitive fitness of *nusG*, *rpoB* and *rpoC* mutants in RifS and βS450L Mtb. (1) Generation of barcode (BC) library; asterisk indicates premature stop codon. (2) ssDNA recombineering to generate mutants of interest. (3) Pooled, competitive growth experiment. Oligo, oligonucleotide. **b**,**c**, Competitive index (mean ± s.d. from 3 biological replicates) of each mutant relative to the RifS base strain (no mutations in *nusG*, *rpoB* or *rpoC*). The competitive index of each mutant was normalized to its respective base strain, RifS or βS450L, by subtracting the competitive index of the base strain. *nusG*-T200A is a control mutation not associated with RifR in clinical isolates and is thus not expected to restore fitness to βS450L Mtb. For reference, the fitness of βS450L relative to RifS in this experiment is 0.96. Paired two-sided *t*-test. **P ≤ 0.01, ***P ≤ 0.001. **d**, Model explaining the mechanism of reduced fitness of βS450L Mtb and its compensation. The βS450L RNAP elongates more slowly and is thus more likely to swivel and enter the paused and termination states. This effect is exacerbated by the pro-pausing wild-type NusG, as illustrated by an increase in the activation barrier of elongation. These interactions decrease βS450L Mtb fitness. Note that the competitive index values shown here are not base-subtracted as they are in **b**,**c**. Compensatory mutations at the NusG–β-protrusion or NusG–NT-DNA interface reduce the ability of NusG to stabilize the swivelled state, as illustrated by a relative decrease in the activation barrier of elongation. These mutations restore βS450L RNAP pausing and termination levels closer to wild-type RNAP and increase βS450L Mtb fitness.

To test the prediction that *nusG* and β-protrusion variants may represent a new form of compensatory evolution to restore fitness in βS450L Mtb, we reconstructed these mutations in isogenic RifS and βS450L Mtb by single-stranded DNA (ssDNA) recombineering (Fig. 5a). These strains also contained unique molecular barcodes, thereby enabling competitive growth experiments and assessment of mutant abundance by deep sequencing. All strains were confirmed by whole-genome sequencing to harbour the targeted mutation and, in the case of βS450L strains, not to harbour untargeted compensatory mutations in *rpoA*, *rpoB* and *rpoC*.

To investigate the effect of these variants on the fitness of RifS and βS450L Mtb, we co-cultured all mutants in a competitive growth experiment, sequenced barcodes to assess mutant abundance, and calculated competitive indices to quantify relative fitness (Fig. 5b,c). As expected, βS450L was less fit than RifS Mtb (Supplementary Table 6). A control *nusG* mutation (T200A) that was found in both RifS and RifR clinical Mtb isolates and thus not expected to be compensatory had no effect on the fitness of βS450L Mtb (Fig. 5c). Consistent with its well-established compensatory behaviour, *rpoC*^F452L^ increased the fitness of βS450L but decreased fitness of RifS Mtb^16,20^. Similarly, all tested *nusG* and β-protrusion variants (except the T200A control) increased the fitness of βS450L but, if anything, decreased fitness of RifS Mtb. Together, these data indicate that *nusG* and β-protrusion variants decrease the pro-pausing and termination activity of NusG and represent a new form of compensatory evolution to restore fitness in βS450L Mtb (Fig. 5d).

## Discussion

It has long been known that RifR produces a fitness cost in bacteria. Here we show that the fitness cost of the most common RifR mutation in Mtb—βS450L—is driven at least in part by excessive RNAP pausing and termination. We further show that mutations in NusG or the β-protrusion reduce RNAP pausing and termination and increase βS450L fitness, and therefore represent a new form of compensatory evolution in RifR Mtb.

We hypothesize that NusG has dual roles in regulating βS450L fitness. Whereas the pro-pausing function of NusG is detrimental for βS450L fitness, another aspect of NusG function promotes βS450L fitness. One way in which NusG could promote βS450L fitness is by mediating TTC. Consistent with a role in translation promoting βS450L fitness, we identify numerous tRNAs, tRNA synthetases, ribosomal proteins, translation factors and amino acid biosynthetic genes as collateral vulnerabilities (Supplementary Table 1). These translation-related collateral vulnerabilities are not differentially expressed at the RNA level and the majority (22 out of 26 detected proteins) are not differentially expressed at the protein level between a panel of paired RifS and βS450L RifR Mtb clinical isolates^27^ (Supplementary Fig. 3a,b). These results argue against a role for pervasive under-expression of translation-related genes as the sole explanation for increased vulnerability in βS450L. That Mtb NusG interacts with the ribosomal protein S10^35^ and the existence of translationally coupled attenuation mechanisms^34,42,43^ suggests that transcription and translation are coupled in Mtb, but direct demonstration of TTC in Mtb remains an important area of future research. Although TTC in Mtb is speculative, it is a reasonable hypothesis that is consistent with the available data. Alternatively, NusG could promote βS450L fitness for other reasons. For example, should Mtb NusG be capable of interacting with Rho, NusG could influence Rho-mediated termination^36^. However, the fact that Rho is not a differential vulnerability (Supplementary Fig. 3c,d) argues against a model in which NusG-stimulated Rho termination is a critical mechanism by which NusG promotes βS450L fitness. The recent Mtb NusG–RNAP structure is potentially consistent with a role for Mtb NusG in preventing RNAP backtracking^13^, which in turn could promote βS450L fitness by promoting RNAP elongation. Although the precise mechanisms by which NusG promotes βS450L fitness require further investigation, our work definitively shows that both the NusG NGN domain and β-protrusion are under selective pressure to mutate to dampen pro-pausing and termination activity to restore βS450L fitness.

Our identification of a genetic association between *nusG* variants and RifR is consistent with other recent work that observed signs of selection in Mtb *nusG*^44,45^. We found that the NusG NGN domain and the RNAP β-protrusion are under positive selection in specific RifR *rpoB* mutants. The vast majority (>95%) of *nusG* or β-protrusion-compensated RifR Mtb strains are βS450L, as is also true for strains harbouring compensatory mutations in *rpoC* or *rpoA*^12^ (Supplementary Fig. 2b and Supplementary Table 2). In a seminal paper, Gagneux and colleagues^15^ performed a laboratory evolution experiment of RifR Mtb to identify mechanisms of fitness compensation. We reanalysed their data and—in addition to their discovery of compensatory mutations in *rpoC* and *rpoA*—we identified a previously overlooked compensatory mutation (T399A) in the β-protrusion. The frequency of compensation by *nusG* or β-protrusion mutations is similar to that seen for *rpoA*, but compensation by *rpoC* mutations is more common (Fig. 3b, Supplementary Fig. 2b and Supplementary Table 2). The higher frequency of compensation by *rpoC* mutations probably reflects at least in part a larger mutational target size in *rpoC* (Supplementary Table 2), but it could also result from more efficient biochemical compensation of the βS450L mutation by secondary mutations in *rpoC*.

Our findings can be understood when considering the recent structures of Mtb RNAP bound to NusG^13^. The bridge helix and the trigger loop are two conserved structural modules in the active site of all cellular RNAPs. Concerted conformational changes of the bridge helix and folding and unfolding transitions of the trigger loop are essential for progression through the nucleotide addition cycle. RNAP swivelling leads to bridge helix distortions that interfere with: (1) the positioning of the DNA base templating the incoming NTP substrate in the active site; and (2) trigger loop folding, which collectively inhibits RNAP elongation. Thus, swivelling is likely to be an off-line RNAP conformation that kinetically competes with normal elongation^39,46^. Whether by cause or effect, the slower elongation rate of the βS450L RNAP is associated with a higher propensity for swivelling, explaining the elevated pause half-lives and termination rates for the mutant RNAP^20^ (Figs. 4c,d and 5d). Mtb NusG promotes RNAP pausing and termination by stabilizing the swivelled conformation^13^. We show here that the βS450L mutation results in selection for additional mutations in either of the two NusG interfaces used to stabilize pausing: the NusG–β-protrusion interface or the NusG–NT-DNA interface (Fig. 5d). These compensatory mutations reduce the stabilizing effects of NusG on swivelling and temper the hyper-termination effects of the βS450L substitution. Thus, increased pausing and termination is at least one source of the fitness cost of βS450L, and one form of compensation is to reduce these activities. Whether compensatory mutations within the RNAP also work by reducing the propensity of βS450L RNAP to swivel and pause and could thus represent a ‘unifying principle’ of βS450L compensation represents an important area of future research.

The hyper-termination phenotype of βS450L RNAP presumably leads to widespread transcriptome changes, but whether expression dysregulation of a few or many genes underlies the fitness cost of βS450L remains an open question. RNA-sequencing and quantitative proteomic studies have shown subtle but pleiotropic differences between the transcriptomes and proteomes of βS450L and RifS Mtb^24,27,47^. One might expect that genes whose under-expression contributes to the fitness cost of βS450L might be regulated by RNAP pausing and termination and be enriched as collateral vulnerabilities in the βS450L screen, a hypothesis that will be tested in future studies. Should such genes be identified, they could in principle serve as additional sources of fitness compensating mutations outside of NusG and RNAP.

RifR RNAP compensatory mutations have been known for more than two decades^12,15,16,19,48^. To our knowledge, our work represents the first biochemically defined mechanism of compensation for RNAP. Intriguingly, compensation works late during the transcription cycle, at the level of transcription elongation. Our work builds on foundational studies that first demonstrated the fitness cost of chromosomal drug-resistance mutations^1,4,5^ and its compensation^1,2,4,7,48–51^. Beyond Rif and RNAP, one of the best studied drug targets is the bacterial ribosome and translation. Several studies in diverse bacteria have shown that resistance to translation-perturbing antibiotics frequently comes with a fitness cost and that there exist diverse mechanisms to compensate for that cost. For example, resistance to aminoglycoside and tuberactinomycin antibiotics can occur through mutations in the 16S ribosomal RNA (rRNA), and compensation can occur by intragenic secondary mutations that restore 16S rRNA secondary structure or non-mutational mechanisms that increase 16S rRNA methylation^8,52,53^. Aminoglycoside resistance can also be caused by mutations in *rpsL*, which encodes the ribosomal protein S12. Although low-cost mutations are by far the most common in clinical Mtb isolates^8^, high-cost mutations can be compensated by a constellation of secondary mutations in *rpsL* or in genes encoding other ribosomal proteins. At least some of these mutations may serve to restore ribosomal translation fidelity altered by the original resistance mutation^4,54^. Peptide deformylase inhibitors target peptide deformylase, a bacterial enzyme that removes the formyl group from the N-terminal methionine in nascent polypeptides. Resistance to peptide deformylase inhibitors commonly occurs by reducing formylation of the methionyl initiator tRNA (Met-tRNAi)—for example, as a result of loss-of-function mutations in formyl-methionine-transferase^55^ (*fmt*). As a consequence of *fmt* mutations, both translation and growth rates are reduced^56^. Compensation for the fitness cost of *fmt* mutants can occur by a myriad of mechanisms, many of which ultimately enable the ribosome to initiate translation without a formylated methionyl initiator tRNA^57–59^. Given these precedents and the evolutionary flexibility of bacteria, it will be important to determine potential fitness costs and mechanisms of compensation for newly developed antibiotics.

The fitness cost of drug resistance serves as the basis for antibiotic combination approaches that aim to limit the evolution and spread of resistant mutants^30^. For example, collateral sensitivity represents an evolutionary trade-off whereby resistance to one antibiotic confers increased sensitivity to another antibiotic. One can imagine several ways in which the findings presented here could be used to this end. For example, the fact that the pro-pausing activity of NusG reduces βS450L fitness suggests that compounds that promote RNAP pausing may be particularly effective in preventing βS450L evolution. Alternatively, small molecule inhibitors of alternative collateral vulnerabilities identified in the screen might serve as good partner drugs with Rif to slow the evolution of βS450L.

We show that excessive pausing and termination contributes to the fitness cost of RifR in Mtb, and that RifR Mtb selects for compensatory mutations in NusG and the β-protrusion that partially alleviate this defect. Compensatory mutations may improve patient-to-patient Mtb transmission^17^, and indeed Mtb clinical strains harbouring mutations in *nusG* were previously associated with increased Mtb transmission in Pakistan^60^. Thus, molecular diagnostic approaches to detect fitness compensated drug-resistant tuberculosis could be used to identify patients with strains that are more likely to be transmitted. Our findings suggest rational approaches to exacerbate the fitness cost of RifR, which could inform new therapeutic approaches to develop drug combinations to slow the evolution of RifR Mtb. Our approach is generalizable to other organisms, setting the stage for expanded differential vulnerability studies to better understand and ultimately target unique vulnerabilities of drug-resistant pathogens.

## Methods

### Bacterial strains

Mtb strains are derivatives of H37Rv unless otherwise noted. Δ*bioA* Mtb was obtained from the Schnappinger laboratory^64^. *E. coli* strains are derivatives of DH5α (NEB), Rosetta2, or BL21(DE3) (Novagen).

### Mycobacterial cultures

Mtb was grown at 37 °C in Difco Middlebrook 7H9 broth or on 7H10 agar supplemented with 0.2% glycerol (7H9) or 0.5% glycerol (7H10), 0.05% Tween-80, 1× oleic acid-albumin-dextrose-catalase (OADC) and the appropriate antibiotics (kanamycin 10–20 μg ml^−1^ and/or hygromycin 25–50 μg ml^−1^). ATc was used at 100 ng ml^−1^. Mtb cultures were grown standing in tissue culture flasks (unless otherwise indicated) with 5% CO_2_. Note that both 7H9 and 7H10 medium are normally supplemented with biotin (0.5 mg l^−1^; ~2 μM), thereby allowing growth of the Δ*bioA* Mtb auxotroph.

### Selection of Rif-resistant Mtb isolates

For the selection of RifR H37Rv and Δ*bioA* Mtb, 5 independent 5-ml cultures were started at a density of ~2,000 cells per ml (to minimize the number of preexisting RifR bacteria) and grown to stationary phase (OD_600_ > 1.5). Cultures were pelleted at 4,000 rpm for 10 min, resuspended in 30 μl remaining medium per pellet and plated on 7H10 agar supplemented with Rif at 0.5 μg ml^−1^. After outgrowth, colonies were picked into 7H9 medium. After 1 week of outgrowth, an aliquot was heat-inactivated and the Rif resistance determining region of *rpoB*, *rpoA* and *rpoC* were amplified by PCR and Sanger sequenced. See Supplementary Table 4 for primer sequences.

### Generation of structural models

The structural model of Mtb RNAP transcription initiation complex bound to Rif in Fig. 1a was generated by modelling *Mycobacterium smegmatis* RNAP bound to Rif (PDB: 6CCV)^65^ on to the transcription initiation complex structure (PDB: 6EDT)^66^.

The cryo-EM structures of a NusG-bound paused elongation complex from Mtb (PDB: 8E74) in Fig. 2d, and the location of clinical isolate mutations in Fig. 4a are derived from Delbeau et al.^13^.

### Generation of individual CRISPRi strains

Individual CRISPRi plasmids were cloned as described^67^ using Addgene plasmid 166886. In brief, the CRISPRi plasmid backbone was digested with BsmBI-v2 (NEB R0739L) and gel-purified. sgRNAs were designed to target the non-template strand of the target gene open reading frame (ORF). For each individual sgRNA, two complementary oligonucleotides with appropriate sticky end overhangs were annealed and ligated (T4 ligase NEB M0202 M) into the BsmBI-digested plasmid backbone. Successful cloning was confirmed by Sanger sequencing.

Individual CRISPRi plasmids were then electroporated into Mtb. Electrocompetent cells were obtained as described^68^. In brief, an Mtb culture was expanded to an OD_600_ = 0.4–0.6 and treated with glycine (final concentration 0.2M) for 24 h before pelleting (4,000*g* for 10 min). The cell pellet was washed three times in sterile 10% glycerol. The washed bacilli were then resuspended in 10% glycerol in a final volume of 5% of the original culture volume. For each transformation, 100 ng plasmid DNA and 100 μl electrocompetent mycobacteria were mixed and transferred to a 2 mm electroporation cuvette (Bio-Rad 1652082). Where necessary, 100 ng plasmid plRL19 (Addgene plasmid 163634) was also added. Electroporation was performed using the Gene Pulser X cell electroporation system (Bio-Rad 1652660) set at 2,500 V, 700 Ω and 25 μF. Bacteria were recovered in 7H9 for 24 h. After the recovery incubation, cells were plated on 7H10 agar supplemented with the appropriate antibiotic to select for transformants.

### CRISPRi library transformation

CRISPRi libraries were generated as described previously^28^. In brief, fifty transformations were performed to generate RifS and βS450L Δ*bioA* libraries. For each transformation, 1 μg of RLC12 plasmid DNA was added to 100 μl electrocompetent cells. The cells:DNA mix was transferred to a 2 mm electroporation cuvette (Bio-Rad 1652082) and electroporated at 2,500 kV, 700 Ω, and 25 μF. Each transformation was recovered in 2 ml 7H9 medium supplemented with OADC, glycerol and Tween-80 (100 ml total) for 16–24 h. The recovered cells were collected at 4,000 rpm for 10 min, resuspended in 400 μl remaining medium per transformation and plated on 7H10 agar supplemented with kanamycin (see ‘Mycobacterial cultures’) in Corning Bioassay dishes (Sigma CLS431111-16EA).

After 21 days of outgrowth on plates, transformants were scraped and pooled. Scraped cells were homogenized by two dissociation cycles on a gentleMACS Octo Dissociator (Miltenyi Biotec 130095937) using the RNA_01 program and 30 gentleMACS M tubes (Miltenyi Biotec 130093236). The library was further declumped by passaging 1 ml of homogenized library into 100 ml of 7H9 supplemented with kanamycin (see Mycobacterial cultures) for between 5 and 10 generations. Final RifS and βS450L Δ*bioA* Mtb library stocks were obtained after passing the cultures through a 10-μm cell strainer (Pluriselect SKU 43-50010-03). Genomic DNA was extracted from the final stocks and library quality was validated by deep sequencing (see ‘Genomic DNA extraction and library preparation for Illumina sequencing’).

### Pooled CRISPRi screen

Pooled CRISPRi screens were performed as described^28^. In brief, 20-ml cultures were grown in vented tissue culture flasks (T-75; Falcon 353136) and 7H9 medium supplemented with kanamycin (see ‘Mycobacterial cultures’) and maintained at 37 °C, 5% CO_2_ in a humidified incubator.

The screen was initiated by thawing 4× 1-ml aliquots of the Mtb Δ*bioA* (RifS or βS450L) CRISPRi library (RLC12) and inoculating each aliquot into 24 ml 7H9 medium supplemented with kanamycin in a T-75 flask (starting OD_600_∼0.06). The cultures were expanded to approximately OD_600_ = 1.0, pooled and passed through a 10-μm cell strainer (pluriSelect 43-50010-03) to obtain a single cell suspension. The single cell suspension (flow-though) was used to set up six ‘generation 0’ cultures: three replicate cultures with ATc (+ATc) and three replicate control cultures without ATc (–ATc). From each generation 0 culture, we collected 10 OD_600_ units of bacteria (∼3 × 10^9^ bacteria; ∼30,000X coverage of the CRISPRi library) for genomic DNA extraction. The remaining culture volume was used to initiate the pooled CRISPRi fitness screen. Cultures were periodically passaged in pre-warmed medium in order to maintain log phase growth. At generation 2.5, 5, and 7.5, cultures were back-diluted 1:6 (to a starting OD_600_ = 0.2) and cultivated for approximately 2.5 doublings. At generation 10, 15, 20, and 25, cultures were back-diluted 1:24 (to a starting OD_600_ = 0.05) and expanded for 5 generations before reaching late-log phase. ATc was replenished at every passage. By keeping the OD_600_ of the 20 ml cultures ≥ 0.05, we guaranteed sufficient coverage of the library (3,000X) at all times. At set time points (approximately 2.5; 5; 7.5; 10; 15; 20; 25 and 30 generations), we collected bacterial pellets (10 OD_600_ units) to extract genomic DNA.

### Genomic DNA extraction and library preparation for Illumina sequencing of CRISPRi libraries

Genomic DNA was isolated from bacterial pellets using the CTAB-lysozyme method described previously^69^. Genomic DNA concentration was quantified using the DeNovix dsDNA high sensitivity assay (KIT-DSDNA-HIGH-2; DS-11 Series Spectrophotometer/Fluorometer).

Illumina libraries were constructed as described^28^. In brief, the sgRNA-encoding region was amplified from 500 ng genomic DNA using NEBNext Ultra II Q5 master Mix (NEB M0544L). PCR cycling conditions were: 98 °C for 45 s; 17 cycles of 98 °C for 10 s, 64 °C for 30 s, 65 °C for 20 s; 65 °C for 5 min. Each PCR reaction a unique indexed forward primer (0.5 μM final concentration) and a unique indexed reverse primer (0.5 μM) (Supplementary Table 4). Forward primers contain a P5 flow cell attachment sequence, a standard Read1 Illumina sequencing primer binding site, custom stagger sequences to ensure base diversity during Illumina sequencing, and unique barcodes to allow for sample pooling during deep sequencing. Reverse primers contain a P7 flow cell attachment sequence, a standard Read2 Illumina sequencing primer binding site, and unique barcodes.

Following PCR amplification, each ∼230 bp amplicon was purified using AMPure XP beads (Beckman–Coulter A63882) using two-sided selection (0.75X and 0.12X). Eluted amplicons were quantified with a Qubit 2.0 Fluorometer (Invitrogen), and amplicon size and purity were quality controlled by visualization on an Agilent 4200 TapeStation (Instrument- Agilent Technologies G2991AA; reagents- Agilent Technologies 5067-5583; tape- Agilent Technologies 5067-5582). Next, individual PCR amplicons were multiplexed into 20 nM pools and sequenced on an Illumina sequencer according to the manufacturer’s instructions. To increase sequencing diversity, a PhiX spike-in of 2.5–5% was added to the pools (PhiX sequencing control v3; Illumina FC-110-3001). Samples were run on the Illumina NextSeq 500 or NovaSeq 6000 platform (single-read 1 ×85 cycles, 8 × i5 index cycles, and 8 × i7 index cycles).

### Differential vulnerability analysis of Rif-resistant versus Rif-sensitive strains

Gene vulnerability in the RifS and βS450L Mtb strains was determined using an updated vulnerability model based on the one previously described^28^. In the updated model, read counts for a given sgRNA in the minus ATc conditions were modelled using a negative binomial distribution with a mean proportional to the counts in the plus ATc condition, plus a factor representing the log_2_ fold change:

$${y}_{i}^{-{\rm{ATc}}} \sim {\rm{NegBinom}}\left({\eta }_{i},\phi \right)$$

$${\eta }_{i}=\log (\,{y}_{i}^{+{\rm{ATc}}}+{\lambda }_{i})+{\rm{TwoLine}}({x}_{i},{\alpha }_{l},{\beta }_{l},\gamma ,{\beta }_{e})$$

where *λ*_*i*_ is an sgRNA-level correction factor estimated by the model, *x*_*i*_ represents the generations analysed for the *i*th guide, and the TwoLine function represents the piecewise linear function previously described, which models sgRNA behaviour over the logistic function describing gene-level vulnerabilities was simplified by setting the top asymptote of the curve (previously *K*) equal to 0, representing the fact that weakest possible sgRNAs are expected to impose no effect on bacterial fitness, that is:

$${\rm{Logistic}}\left(s\right)=\frac{{\beta }_{\max }}{\left(1+{{\rm{e}}}^{\left(-H\cdot \left(s-M\right)\right)}\right)}$$

The Bayesian vulnerability model was run for each condition independently, and samples for all the parameters were obtained using Stan running 4 independent chains with 1,000 warmup iterations and 3,000 samples each (for a total of 12,000 posterior samples for each parameter in the model after discarding warmup iterations).

Differential vulnerabilities were estimated by two approaches. First, for each gene, the difference in pairwise (guide-level) vulnerability estimates was obtained, resulting in posterior samples of the differential vulnerability (delta-vulnerability). This effectively estimated the difference in the integrals of the vulnerability functions. If the 95% credible region did not overlap 0.0 those were taken as significant differential vulnerabilities between the strains.

Next, to identify differences between genes which may not exhibit the expected dose–response curve, we estimated the fitness cost (log_2_FC) predicted by our model for a (theoretical) sgRNA of strength 0.0 (that is, Logistic(*s* = 0)). This represented the weakest phenotype theoretically possible with our CRISPRi system, which we call *F*_min_. The difference between this value was estimated for each gene (∆*F*_min_) and those where the 95% credible region did not overlap 0.0 were identified as significant differential vulnerabilities by this approach.

### Pathway analysis

First, all annotated Mtb genes were associated with a pathway as defined by the Kyoto Encyclopedia of Genes and Genomes (KEGG) database^70–72^. If necessary, annotations were manually curated to update or correct pathway assignments. To quantify pathway enrichment, the query set was defined as the union of the upper quartile of differential vulnerabilities defined by both the original gene vulnerability calling method (Δ*V*) and the *F*_min_ approach. The background set was defined as all annotated Mtb genes. Enrichment of the pathways identified as differentially vulnerable was calculated by an odds ratio and significance was determined with a Fisher’s exact test.

### phyOverlap

To detect associations between gene variants and Rif resistance, we employed a phylogenetic convergence test using the phyOverlap algorithm^73^ (https://github.com/Nathan-d-hicks/phyOverlap). In brief, FASTQ files were aligned to H37Rv genome (NC_018143.2) using bwa (version 0.7.17-r1188). FASTQ accession numbers are provided in Supplementary Table 3. Single-nucleotide polymorphisms (SNPs) were called and annotated using the HaplotypeCaller tool Genome Analysis Toolkit (version 3.5) using inputs from samtools (version 1.7). SNP sites with less than 10x coverage or missing data in >10% strains were removed from the analysis. Repetitive regions of the genome (PE/PPE genes, transposases, and prophage genes) are excluded from the analysis. Known drug-resistance regions were further excluded so as not to bias phylogenetic tree construction. *M. canetti* was provided as an outgroup (NC_015848). We performed Maximum Likelihood Inference using RAxML (v8.2.11) to construct the ancestral sequence and determine the derived state of each allele. Overlap with Rif resistance was scored by dividing the number of genotypically predicted (Mykrobe v0.9.012) RifR isolates containing a derived allele by the total number of isolates with a derived allele at a given genomic position. To generate a gene-wide score, we excluded synonymous SNPs and averaged the individual nonsynonymous SNP scores, weighting the scores by the number of times derived alleles evolved across the phylogenetic tree. The significance of the overlap is then tested by redistributing mutation events for each SNP randomly across the tree and recalculating the score. This permutation is done 50,000 times to derive the *P* value. This analysis additionally used FastTree (version 2.1.11) and figTree (v1.4.4).

### dN/dS calculations

The ratio of nonsynonymous (dN) to synonymous (dS) nucleotide substitutions was used to quantify selective pressure acting on *nusG* and *rpoC*. A dN/dS value less than one suggests negative or purifying selection whereas a dN/dS value greater than one suggests positive or diversifying selection. For this analysis, we used a collection of ~50,000 Mtb clinical isolate whole-genome sequences, as described^41^. Isolates were grouped based on the presence of genotypically predicted Rif resistance (Mykrobe v0.9.012), as well as the identity of the *rpoB* mutation (S450X or H445X; where X indicates any amino acid other than Ser or His, respectively) conferring RifR. The number of samples used in the *nusG* dN/dS analysis shown in Fig. 3 are as follows: 1,365 RifS, 350 RifR, 270 S450X, and 26 H445X. The number of samples used in the *rpoC* dN/dS analysis shown in Fig. 3 are as follows: 23,024 RifS, 13,993 RifR, 11,067 S450X, and 1,215 H445X. Insertions and deletions were necessarily excluded from this analysis. A bootstrap-analysis was performed to calculate the dN/dS ratios to reduce any potential effects of recent clonal expansion events or convergent evolution of a specific site, like acquired drug-resistance mutations, as performed previously^44^. The analysis was performed by sub-sampling 80% of total variants in each group. The sub-sampling was repeated 100 times. dN/dS values were calculated for each subset of samples using a python script obtained from the github repository: https://github.com/MtbEvolution/resR_Project/tree/main/dNdS.

### SNP calling and upset plot

SNP information for all Mtb clinical isolate whole-genome sequences were called as follows. FASTQ reads were aligned to the H37Rv genome (NC_018143.2) and SNPs were called and annotated using Snippy9 (version 3.2-dev) using default parameters (minimum mapping quality of 60 in BWA, samtools base quality threshold of 20, minimum coverage of 10, minimum proportion of reads that differ from reference of 0.9). Mapping quality and coverage was further assessed using QualiMap with the default parameters (version 2.2.2-dev). Samples with a mean coverage < 30, mean mapping quality ≤ 45, or GC content ≤ 50% or ≥ 70% were excluded. Drug resistance-conferring SNPs were annotated using Mykrobe (v0.9.012). The resulting SNP and drug-resistance calls were used to generate the values depicted in the upset plot.

### Phylogenetic trees

Phylogenetic trees based on SNP calls described above were built using FastTree (version 2.1.11 SSE3). A list of SNPs in essential genes was concatenated to build phylogenetic trees. Indels, drug resistance-conferring SNPs, and SNPs in repetitive regions of the genome (PE/PPE genes, transposases and prophage genes) were excluded. Tree visualization was performed in iTol (https://itol.embl.de/).

### Barcode library production

The barcode library was designed to include over 100,000 random 18-mer sequences cloned into an Giles-integrating backbone (*attP* only, no Integrase) containing a hygromycin resistance cassette with a premature stop codon (plNP472). Oligonucleotides were synthesized as a gBlocks Library by IDT, containing 104,976 fragments.

plNP472 (1.6 μg) was digested with PciI (NEB R0655) and gel-purified (QIAGEN 28706). The library was PCR amplified using NEBNext High-Fidelity 2X PCR Master Mix (NEB M0541L). One 50-μl reaction was prepared, containing 25 μl of PCR master mix, 0.0125 pmol of the gBlock library, and a final concentration of 0.5 μM of the appropriate forward and reverse primers (Fwd: 5′-TTACGCGTTTCACTGGCCGATTG-3′ + Rev: 5′-TTTTGCTGGCCTTTTGCTCAAC-3′). PCR cycling conditions were: 98 °C for 30 s; 15 cycles of 98 °C for 10 s, 68 °C for 10 s, 72 °C for 15 s; 72 °C for 120 s. The PCR amplicon were purified using the QIAGEN MinElute PCR purification kit (QIAGEN 28004). One Gibson assembly reaction (NEB E2621) was prepared with 0.01 pmol μl^−1^ digested plNP472 backbone, 0.009 pmol μl^−1^ cleaned PCR amplicon, and master mix, representing a 1:2 molar ratio of vector:insert.

Following incubation at 50 °C for 1 h, 7 μl the Gibson product was dialysed to remove salts and transformed into 100 μl MegaX DH10B T1R Electrocomp Cells (Invitrogen C640003) diluted with 107 μl 10% glyerol. For each of three total transformations, 75 μl of the cells:DNA mix was transferred to a 0.1 cm electroporation cuvette (Bio-Rad 1652089) and electroporated at 2,000 V, 200 ohms, 25 μF. Transformations were washed twice with 300 μl provided recovery medium and recovered in a total of 3 ml medium. Cells were allowed to recover at 37 °C with gentle rotation. Recovered cells were plated across three plates of LB agar supplemented with zeocin. After 1 d incubation at 37 °C, transformants were scraped and pooled. One fourth of the pellet (3.2 g dry mass) was used to perform 24 minipreps using a QIA prep Spin Miniprep Kit (Qiagen 27104).

### Transformation of barcode library into Mtb

The barcode library was transformed into RifS and βS450L Mtb expressing RecT (mycobacteriophage recombinase) similarly to the CRISPRi library (see CRISPRi library transformation), with minor modifications. In brief, cultures for competent cells were grown in 7H9 supplemented with kanamycin to retain the episomal *recT* encoding plasmid (plRL4). Twenty-millilitre cultures were concentrated ten times and transformed with 250 ng of library and 100 ng of non-replicating, Giles integrase containing plasmid (plRL40). Additionally, after recovery cells were plated on 7H10 agar supplemented with kanamycin and zeocin. Transformants were scrapped after 29 days of outgrowth.

### ssDNA recombineering and validation of strains

Clinical *nusG*, *rpoB* and *rpoC* mutants were introduced into RifS and βS450L Mtb using oligonucleotide-mediated (ssDNA) recombineering, as described previously^68^. In brief, 70-mer oligonucleotides were designed to correspond to the lagging strand of the replication fork, with the desired mutation in the middle of the sequence. Alterations were chosen to avoid recognition by the mismatch-repair machinery of RecT expression was induced ~16 h before transformation by addition of ATc to a final concentration of 0.5 μg ml^−1^. 400 μl of competent cells were transformed with 5 μg of mutation containing oligonucleotide and 0.1 μg of hygromycin resistance cassette repair oligonucleotide (1:50 ratio of mutant oligonucleotide to repair oligonucleotide) and recovered in 5 ml 7H9 medium.

After 24 h of recovery, 200 μl of cells were plated on 7H10 plates supplemented with hygromycin. After 21 days of outgrowth, 12 colonies per construct were picked into 100 μl 7H9 medium supplemented with hygromycin in a 96 well plate (Fischer Scientific 877217). 50 μl of culture were heat-inactivated at 80 °C for 2 h in a sealed microamp 96 well plate (Fischer Scientific 07200684; Applied Biosystems N8010560). Fifty microlitres of heat-inactivated culture was mixed with 50 μl of 25% DMSO and lysed at 98 °C 10 min.

Mutations of interest and unique barcodes were confirmed with PCR amplification and Sanger sequencing. The region of interest was PCR amplified with NEBNext High-Fidelity 2X PCR Master Mix (NEB M0541L) using 0.5 μl of heat-lysed product with the appropriate primers, annealing temperatures and extension times (see Supplementary Table 4). Residual PCR primers were removed with NEB Shrimp Alkaline Phosphatase (rSAP) and exonuclease I (exo) (rSAP- NEB M0371; exo- NEB M0293) per manufacturer’s instructions. Amplicons were then submitted for Sanger sequencing. One to three unique independent isolates were generated for all tested mutations.

### Pooled barcode competitive growth assay

Validated mutants were first grown in 1 ml 7H9 with hygromycin and after 3 days, expanded to 5 ml 7H9 with hygromycin. Strains were pooled to contain approximately 1.2 × 10^7^ cells for each mutant. The pool was then diluted to a starting OD_600_ of 0.01 in 7H9 supplemented with hygromycin. At this point, three 20 ml cultures in vented tissue culture flasks (T-75; Falcon 353136) were expanded to late-log phase and used as input for the competitive growth experiment. Sixteen OD_600_ units of cells were collected from flask as the input culture (generation 0). Triplicate cultures were then diluted back to OD_600_ = 0.05 and grown for ~4.5 generations, back-diluted again to OD_600_ = 0.05 and grown for an additional 4 generations. After this, cultures were collected for a cumulative 8.5 generations of competitive growth.

Genomic DNA extraction and library preparation for next-generation sequencing followed the same protocol as that of the CRISPRi libraries (see above), with minor modifications. In brief, the barcode region was amplified from 100 ng genomic DNA using NEBNext Ultra II Q5 master Mix (NEB M0544L). PCR cycling conditions were: 98 °C for 45 s; 16 cycles of 98 °C for 10 s, 64 °C for 30 s, 65 °C for 20 s; 65 °C for 5 min. Each PCR reaction contained a unique indexed forward primer (0.5 μM final concentration) and a unique indexed reverse primer (0.5 μM) (see Supplementary Table 4). Additionally, individual PCR amplicons were multiplexed into a 1 nM pool and sequenced on an Illumina sequencer according to the manufacturer’s instructions. To increase sequencing diversity, a PhiX spike-in of 20% was added to the pool (PhiX sequencing control v3; Illumina FC-110-3001). Samples were run on the Illumina MiSeq Nano platform (paired-read 2 ×150 cycles, 8 × i5 index cycles, and 8 × i7 index cycles).

### WGS and SNP calling for passaging timepoints and ssDNA recombinants

Genomic DNA (gDNA) was extracted as described above. gDNA was diluted and subjected to Illumina whole-genome sequencing by SeqCenter. In brief, Illumina libraries were generated through tagmentation-based and PCR-based Illumina DNA Prep kit and custom IDT 10 bp unique dial indices, generating 320 bp amplicons. Resulting libraries were sequenced on the Illumina NovaSeq 6000 platform (2 × 150 cycles). Demultiplexing quality control, and adapter trimming was performed with bcl-convert (v4.1.5).

Reads were aligned to the Mtb (H37Rv; CP003248.2) reference genome using bwa (v1.3.1) with default parameters. Variant detection was performed by Snippy (v4.6.0)/freebayes (v1.3.1). Resulting vcf files were inspected for compensatory mutations (Supplementary Table 2) in *rpoABC* and/or the presence of the desired mutation.

### Definition of putative compensatory *nusG, rpoA, rpoB, rpoC* variants

Compensatory mutations in *rpoA*, *rpoB* and *rpoC* were taken from published sources and are described in Supplementary Table 2. Inclusion as a putative compensatory mutation in our list required that each reported variant in *rpoA*, *rpoB*, or *rpoC* was found specifically in Rif-resistant strains, defined here as meaning that ≥90% of all strains harbouring the putative compensatory mutation were genotypically predicted (gDST) RifR. The use of the ≥90% gDST RifR cut-off allows for presumptive instances of incorrect gDST calls for strains harbouring rare compensatory variants. The strains used for this analysis are the approximately 50,000 Mtb WGS strain collection described previously^41^.

The rules to define putative compensatory *nusG* mutations are as follows. Each *nusG* variant observed was assessed according to the following three rules and, if it met one of them, was deemed a putative compensatory variant.

1. The *nusG* variant was found in ≥80% genotypically predicted (gDST) RifR strains and was present in at least two distinct Mtb (sub)lineages. The use of the ≥80% gDST RifR cut-off allows for presumptive instances of incorrect gDST calls for strains harbouring rare *nusG* variants.
2. The *nusG* variant was found in 100% gDST RifR strains but only present in a single Mtb sublineage, but the same or nearby NusG site (±5 amino acids) was also mutated to an alternative amino acid that met the criteria stated in rule 1.
3. Residues based on the Mtb NusG–RNAP structure^13^ that were predicted to be important for the NusG pro-pausing activity (for example, NusG Trp120).

The rules to define a putative compensatory mutation in the *rpoB* β-protrusion were similar to those described for *nusG*, except that only *rpoB* β-protrusion residues at or near the NusG interface (RpoB Arg392–Thr410) were included in the analysis. Note that two such β-protrusion mutations (Thr400Ala and Gln409Arg) were previously identified as putative compensatory mutation^17,74,75^ (Supplementary Table 2).

### RifR *rpoB* allele frequency distribution calculations

To check whether the observed distribution of RifR *rpoB* mutations was different for each of the three groups (all RifR strains in our clinical strain genome database, those harbouring known compensatory mutations in *rpoA* or *rpoC*, or those harbouring compensatory mutations in *nusG* or the β-protrusion), we performed a chi-squared test on the observed RifR *rpoB* mutant frequencies. Specifically, we take the RifR *rpoB* mutant frequencies observed in all RifR samples as representing an estimate of the base probabilities under the null hypothesis. We then use these base probabilities to calculate the frequency of mutations that would be expected in the other groups, based on the null hypothesis. That is:

For each mutation (*m*):

$$p(m)=\frac{{\rm{Number}}\,{\rm{of}}\,{\rm{times}}\,m\,{\rm{occurs}}\,{\rm{in}}\,{\rm{RifR}}\,{\rm{samples}}}{{\rm{Total}}\,{\rm{number}}\,{\rm{of}}\,{\rm{RifR}}\,{\rm{samples}}}$$

For each group (*G*) and mutation (*m*),

$$E\left[m| G\right]=p\left(m\right)\times {\rm{total}}\,{\rm{number}}\,{\rm{of}}\,{\rm{samples}}\,{\rm{in}}\,G$$

### Protein expression and purification

#### Mtb RNAP

Mtb RNAP was purified as previously described^66,76^. In brief, plasmid pMP61 (wild-type RNAP) or pMP62 (S450L RNAP) was used to overexpress *Mtb* core RNAP subunits *rpoA*, *rpoZ*, a linked *rpoBC* and a His_8_ tag. pMP61/pMP62 was grown in *E. coli* Rosetta2 cells in LB with 50 μg ml^−1^ kanamycin and 34 μg ml^−1^ chloramphenicol at 37 °C to an OD_600_ of 0.3, transferred to room temperature and left shaking to an approximate OD_600_ of 0.6. RNAP expression was induced by adding IPTG to a final concentration of 0.1 mM, grown for 16 h, and collected by centrifugation (8,000*g*, 15 min at 4 °C). Collected cells were resuspended in 50 mM Tris-HCl, pH 8.0, 1 mM EDTA, 1 mM PMSF, 1 mM protease inhibitor cocktail, 5% glycerol and lysed by sonication. The lysate was centrifuged (27,000*g*, 15 min, 4 °C) and polyethyleneimine (PEI, Sigma-Aldrich) added to the supernatant to a final concentration of 0.6% (w/v) and stirred for 10 min to precipitate DNA binding proteins including target RNAP. After centrifugation (11,000*g*, 15 min, 4 °C), the pellet was resuspended in PEI wash buffer (10 mM Tris-HCl, pH 7.9, 5% v/v glycerol, 0.1 mM EDTA, 5 mM DTT, 300 mM NaCl) to remove non-target proteins. The mixture was centrifuged (11,000*g*, 15 min, 4 °C), supernatant discarded, then RNAP eluted from the pellet into PEI Elution Buffer (10 mM Tris-HCl, pH 7.9, 5% v/v glycerol, 0.1 mM EDTA, 5 mM DTT, 1 M NaCl). After centrifugation, RNAP was precipitated from the supernatant by adding (NH_4_)_2_SO_4_ to a final concentration of 0.35 g l^−1^. The pellet was dissolved in Nickel buffer A (20 mM Tris pH 8.0, 5% glycerol, 1 M NaCl, 10 mM imidazole) and loaded onto a HisTrap FF 5 ml column (GE Healthcare Life Sciences). The column was washed with Nickel buffer A and then RNAP was eluted with Nickel elution buffer (20 mM Tris, pH 8.0, 5% glycerol, 1 M NaCl, 250 mM imidazole). Eluted RNAP was subsequently purified by gel filtration chromatography on a HiLoad Superdex 26/600 200 pg in 10 mM Tris pH 8.0, 5% glycerol, 0.1 mM EDTA, 500 mM NaCl, 5 mM DTT. Eluted samples were aliquoted, flash frozen in liquid nitrogen and stored in −80 °C until usage.

#### Mtb σ^A^–RbpA

Mtb σA–RbpA was purified as previously described^76,77^. The Mtb σA expression vector pAC2 contains the T7 promoter, ten histidine residues, and a precision protease cleavage site upstream of Mtb σA. The Mtb RbpA vector is derived from the pET-20B backbone (Novagen) and contains the T7 promoter upstream of untagged Mtb RbpA. Both plasmids were co-transformed into *E. coli* Rosetta2 cells and selected on medium containing kanamycin (50 µg ml^−1^), chloramphenicol (34 µg ml^−1^) and ampicillin (100 µg ml^−1^). Protein expression was induced at OD_600_ of 0.6 by adding IPTG to a final concentration of 0.5 mM and leaving cells to grow at 30 °C for 4 h. Cells were then collected by centrifugation (4,000*g*, 20 min at 4 °C). Collected cells were resuspended in 50 mM Tris-HCl, pH 8.0, 500 mM NaCl, 5 mM imidazole, 0.1 mM PMSF, 1 mM protease inhibitor cocktail, and 1 mM β-mercaptoethanol, then lysed using a continuous-flow French press. The lysate was centrifuged twice (15,000*g*, 30 min, 4 °C) and the proteins were purified by Ni^2+^-affinity chromatography (HisTrap IMAC HP, GE Healthcare Life Sciences) via elution at 50 mM Tris-HCl, pH 8.0, 500 mM NaCl, 500 mM imidazole, and 1 mM β-mercaptoethanol. Following elution, the complex was dialysed overnight into 50 mM Tris-HCl, pH 8.0, 500 mM NaCl, 5 mM imidazole, and 1 mM β-mercaptoethanol and the His_10_ tag was cleaved with precision protease overnight at a ratio of 1:30 (protease mass:cleavage target mass). The cleaved complex was loaded onto a second Ni^2+^-affinity column and was retrieved from the flow-through. The complex was loaded directly onto a size-exclusion column (SuperDex-200 16/16, GE Healthcare Life Sciences) equilibrated with 50 mM Tris-HCl, pH 8, 500 mM NaCl, and 1 mM DTT. The sample was concentrated to 4 mg ml^−1^ by centrifugal filtration and stored at –80 °C until usage.

#### Mtb CarD

Mtb CarD was purified as previously described^66,76^. In brief, Mtb CarD was overexpressed from pET SUMO (Invitrogen) in *E. coli* BL21(DE3) cells (Novagen) and selected on medium containing 50 µg ml^−1^ kanamycin. Protein expression was induced by adding IPTG to a final concentration of 1 mM when cells reached an apparent OD_600_ of 0.6, followed by 4 h of growth at 28 °C, then collected by centrifugation (4,000*g*, 15 min at 4 °C). Collected cells were resuspended in 20 mM Tris-HCl, pH 8.0, 150 mM potassium glutamate, 5 mM MgCl_2_, 0.1 mM PMSF, 1 mM protease inhibitor cocktail, and 1 mM β-mercaptoethanol, then lysed using a continuous-flow French press. The lysate was centrifuged twice (16,000*g*, 30 min, 4 °C) and the proteins were purified by Ni^2+^-affinity chromatography (HisTrap IMAC HP, GE Healthcare Life Sciences) via elution at 20 mM Tris-HCl, pH 8.0, 150 mM potassium glutamate, 250 mM imidazole, and 1 mM β-mercaptoethanol. Following elution, the complex was dialysed overnight into 20 mM Tris-HCl, pH 8.0, 150 mM potassium glutamate, 5 mM MgCl_2_, and 1 mM β-mercaptoethanol and the His_10_ tag was cleaved with ULP-1 protease (Invitrogen) overnight at a ratio of 1/30 (protease mass/cleavage target mass). The cleaved complex was loaded onto a second Ni^2+^-affinity column and was retrieved from the flow-through. The complex was loaded directly onto a size-exclusion column (SuperDex-200 16/16, GE Healthcare Life Sciences) equilibrated with 20 mM Tris-HCl, pH 8, 150 mM potassium glutamate, 5 mM MgCl_2_ and 2.5 mM DTT. The sample was concentrated to 5 mg ml^−1^ by centrifugal filtration and stored at –80 °C.

#### Wild-type Mtb NusG (+ mutants N65H, R124L and N125S)

Plasmid pAC82 (or mutant variation) was used to overexpress wild-type Mtb NusG^13^. Plasmids encoding NusG mutants were generated using Q5 Site-directed mutagenesis (NEB) and sequenced to confirm the presence of target mutations. *E. coli* BL21 cells containing plasmids encoding different versions of Mtb NusG were grown in LB with 50 μg ml^−1^ kanamycin at 37 °C to an OD_600_ of 0.4, then transferred to room temperature and left shaking to an OD_600_ of 0.67. Protein expression was induced by adding IPTG to a final concentration of 0.1 mM, grown for an additional 4 h, then collected by centrifugation (4,000*g*, 20 min at 4 °C). Collected cells were resuspended in 50 mM Tris-HCl, pH 8.0, 500 mM NaCl, 5 mM imidazole, 10% glycerol, 1 mM PMSF, 1 mM protease inhibitor cocktail (Roche), 2 mM β-mercaptoethanol, and lysed by French press. The lysate was centrifuged (4,000 rpm for 20 min, 4 °C) and the supernatant was removed and applied to a HisTrap column pre-washed with 50 mM Tris-HCl, pH 8.0, 500 mM NaCl, 10% glycerol, 15 mM imidazole, and 2 mM β-mercaptoethanol. After loading the sample, the column was washed with five volumes of the same buffer, before gradient elution with 50 mM Tris-HCl, pH 8.0, 500 mM NaCl, 10% glycerol, 250 mM imidazole, and 2 mM β-mercaptoethanol. The eluted protein was mixed with precision protease and dialysed overnight at 4 °C in 20 mM Tris-HCl, pH 8.0, 500 mM NaCl, 10 mM β-mercaptoethanol to cleave the N-terminal His_10_ tag before applying to a HisTrap column to remove the uncleaved protein. The flow-through was collected and glycerol was added to a final concentration of 20% (v/v). Aliquots were flash frozen in liquid nitrogen and stored in –80 °C until use.

#### Promoter-based in vitro termination assays

The DNA sequence for the Mtb H37Rv 5 S rRNA (*rrf* gene) intrinsic terminator was taken from Mycobrowser (MTB000021), with genomic coordinates of 1,476,999 to 1,477,077 basepairs. The intrinsic terminator was found by predicting its RNA structure using mfold (RNA folding form v2.3) via the UNAFold Web Server. The intrinsic terminator was cloned downstream of a cytidine-less halt cassette in plasmid pAC70^38^, a gift of the R. Landick laboratory, using Q5 site-directed mutagenesis (following manufacturer’s protocol – NEB) at an annealing temperature of 59 °C with GC enhancer for the PCR step, with primers 5′-TGGTGTTTTTGTATGTTTATATCGACTCAGCCGCTCGCGCCATGGACGCTCTCCTGA-3′ and 5′-CCGTTACCGGGGGTGTTTTTGTATGTTCGGCGGTGTCCTGGATCCTGGCAGTTCCCT-3′ (synthesized by IDT), to create plasmid pJC1. The 323 base pairs linear DNA fragment used for in vitro transcription assays was PCR amplified using Accuprime Pfx DNA polymerase (Invitrogen) at an annealing temperature of 56.5 °C, with primers 5′-GAATTCAAATATTTGTTGTTAACTCTTGACAAAAGTGTTAAAAGC-3′ and 5′-GTTGCTTCGCAACGTTCAAATCC-3′ (synthesized by IDT), following manufacturer instructions, and PCR purified (using the QIAquick PCR Purification Kit (QIAGEN)) to remove protein contents and buffer exchange into 10 mM Tris-HCl pH 8.5.

pJC1 contains the *rrf* termination site at approximately +150 bp. This template also contained a C-less cassette (+1 to +26). Core RNAP was incubated for 15 min at 37 °C with σA/RbpA in transcription buffer (20 mM Tris, 25 mM KGlu, 10 mM MgOAc, 1 mM DTT, 5 µg ml^−1^ BSA) to form holo-RNAP, followed by 10 min incubation with 500 nM CarD at 37 °C. Holo-RNAP (200 nM) was then incubated with template DNA (10 nM) for 15 min at 37 °C. To initiate transcription, the complex was incubated with ATP + GTP (both at 16 µM), UTP (2 µM), and 0.1 µl per reaction [α-^32^P]UTP for 15 min at 37 °C to form a halted complex at U26. Transcription was restarted by adding a master mix containing NTP mix (A + C + G + U), heparin, and NusG at a final concentration of 150 µM (each NTP), 10 µg ml^−1^ (heparin), and 1 µM NusG at 23 °C. The reaction was allowed to proceed for 30 min, followed by a ‘chase’ reaction in which all 4 nucleotides were added to a final concentration of 500 µM each. After 10 min, aliquots were removed and added to a 2× Stop buffer (95% formamide, 20 mM EDTA, 0.05% bromophenol blue, 0.05% xylene cyanol). Samples were analysed on an 8% denaturing PAGE (19:1 acrylamide: bis acrylamide, 7 M urea, 1X TBE pH= 8.3) for 1.25 h at 400 V, and the gel was exposed on a Storage Phosphor Screen and imaged using a Typhoon PhosphoImager (GE Healthcare).

#### Quantification of termination and changes in termination

Synthesized RNA bands on the gel image were quantified using ImageJ software (NIH). Each lane from below the *rrf* termination site (~150 nt) to above the runoff RNA products (263 nt) was converted to a pseudo-densitometer plot using the ImageJ line function and the relative areas of the termination and runoff bands were measured. Termination efficiency (TE) was calculated as the fraction of the termination (term) peak area relative to total of the termination and runoff (term + runoff) peak areas. Fold changes in termination attributable to each NusG (∆*T*) were determined as the aggregate of changes in the termination rates *k*_b_ and *k*_t_, as defined by von Hippel and Yager (equations (1) and (2))^62,63^. Multiple algebraic transforms can yield the aggregate fold changes in termination, ∆*T*, based on the following equations.
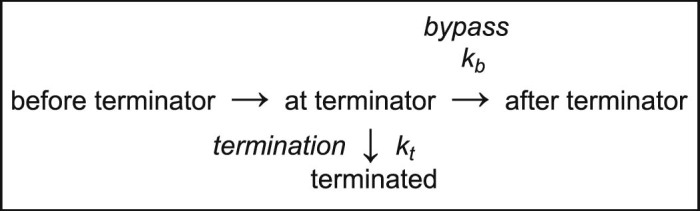

1 $${\rm{TE}}=\frac{{k}_{t}}{{k}_{t}+{k}_{b}}$$

2 $${\rm{TE}}={\left[1+{{\rm{e}}}^{-\Delta \Delta {G}^{\ddagger }/-RT}\right]}^{-1},$$

where ∆∆*G*^‡^ is the difference in activation barriers between termination and bypass, which is most directly related to the energies of RNAP–NusG and internal RNAP interactions that govern termination.

3 $${\Delta \Delta G}^{\ddagger }=-RT\times {\rm{ln}}\left(\left(1/{\rm{TE}}\right)-1\right)$$

(equation (2) rearranged).

4 $$\Delta T={{\rm{e}}}^{\left({\Delta \Delta G}_{2}^{\ddagger }-{\Delta \Delta G}_{1}^{\ddagger }\right)}$$

(fold change in aggregate termination rates for two conditions, 1 and 2).

5 $$\Delta T=\frac{\left(\frac{1}{{{\rm{TE}}}_{2}}\right)-1}{\left(\frac{1}{{{\rm{TE}}}_{1}}\right)-1}$$

(alternative calculation derived from equation (1) assuming NusG only affects *k*_b_).

Calculating ∆*T* using either the combinations of equations (3) and (4) or using equation (5) gives the same results because the ∆*T* is the same whether conditions differ by aggregate effects on both *k*_b_ and *k*_t_ or an effect on only one of them. We calculate ∆*T* using these approaches rather than the simple difference in energies of activation ($\Delta \Delta {G}_{2}^{\ddagger }-\Delta \Delta {G}_{1}^{\ddagger }$) because it allows a clearer graphical depiction of effects without changing the results. Errors in ∆*T* were calculated using a two-sided, unpaired *t*-test with no assumptions on variance.

#### Electrophoretic mobility shift assay

RNAP–NusG complexes were assembled and run on an electrophoretic mobility shift assay to test proper binding of all mutant NusGs. Core RNAP (200 nM) was incubated with the template strand of elongation scaffold DNA^13^ (50 nM) for 15 min at room temperature. Next, the complex was incubated with the complementary non-template strand (50 nM) for 15 min at room temperature. Finally, the complex was incubated with 1 µM wild-type NusG, N65H NusG, R124L NusG, or N125S NusG for 10 min at room temperature. All complexes were assembled in the following transcription buffer: 20 mM Tris, 25 mM potassium glutamate, 10 mM magnesium acetate, 1 mM DTT, 5 µg ml^−1^ BSA. Samples were immediately loaded and run on a native PAGE (4.5% acrylamide:bis solution 37.5:1, 4% glycerol, 1× TBE) for 1 h at 15 mA. The gel was run at 4 °C. The gel was first stained with GelRed (Biotium) followed by Coomassie blue for visualization of DNA and protein respectively.

### Reporting summary

Further information on research design is available in the Nature Portfolio Reporting Summary linked to this article.

## Online content

Any methods, additional references, Nature Portfolio reporting summaries, source data, extended data, supplementary information, acknowledgements, peer review information; details of author contributions and competing interests; and statements of data and code availability are available at 10.1038/s41586-024-07206-5.

## Supplementary information

Supplementary Methods

Reporting Summary

Supplementary Figures Supplementary Figs. 1–4.

Supplementary Table 1 Differential vulnerability results between RifR (RR; BetaS450L) and Rif-sensitive (RS) *M. tuberculosis*.

Supplementary Table 2 Compensatory mutations.

Supplementary Table 3 Variants observed in clinical strain database.

Supplementary Table 4 Plasmids and primers used in this work.

Supplementary Table 5 phyOverlap results.

Supplementary Table 6 Competitive indexes.

## Data Availability

Raw sequencing data are deposited to the NCBI Short Read Archive under project number PRJNA1021243. The H37Rv reference genome (CP003248.2) was applied for alignments and SNP calling. Manually curated pathway calls were derived from KEGG (https://www.genome.jp/kegg-bin/show_organism?org=mtu) and PATRIC databases (https://www.bv-brc.org/search/?keyword(tuberculosis)) databases.

## Code availability

All source code is available at https://github.com/rock-lab/nusg_paper_2023.
