# Supplementary material for: Compensatory evolution in NusG improves fitness of drug-resistant M. tuberculosis
Source: Nature. 2024 Mar 20;628(8006):186–94. doi: 10.1038/s41586-024-07206-5 (PMC10990936; doi:10.1038/s41586-024-07206-5)
Supplement: Supplementary file 1 — Supplementary Methods [file 41586_2024_7206_MOESM1_ESM.pdf]

nature portfolio

https://doi.org/10.1038/s41586-024-07206-5

Supplementary information

Compensatory evolution in NusG improves fitness of drug-resistant *M. tuberculosis*

In the format provided by the authors and unedited

Supplementary methods:

Bacterial strains

Mtb strains are derivatives of H37Rv unless otherwise noted. Δ*bioA* Mtb was obtained from the Schnappinger lab[64]. *E. coli* strains are derivatives of DH5alpha (NEB), Rosetta2, or BL21(DE3) (Novagen).

Mycobacterial cultures

Mtb was grown at 37°C in Difco Middlebrook 7H9 broth or on 7H10 agar supplemented with 0.2% glycerol (7H9) or 0.5% glycerol (7H10), 0.05% Tween-80, 1X oleic acid-albumin-dextrose-catalase (OADC) and the appropriate antibiotics (kanamycin 10–20 µg ml−1 and/or hygromycin 25-50 µg ml−1). Anhydrotetracycline (ATc) was used at 100 ng ml−1. Mtb cultures were grown standing in tissue culture flasks (unless otherwise indicated) with 5% CO2. Note that both 7H9 and 7H10 media are normally supplemented with biotin (0.5 mg/L; ~2 µM), thereby allowing growth of the Δ*bioA* Mtb auxotroph.

Selection of rifampicin-resistant Mtb isolates

For the selection of RifR H37Rv and Δ*bioA* Mtb, five independent 5 mL cultures were started at a density of ~2,000 cells/mL (to minimize the number of preexisting RifR bacteria) and grown to stationary phase (OD600 > 1.5). Cultures were pelleted at 4,000 rpm for 10 minutes, resuspended in 30 µL remaining media per pellet and plated on 7H10 agar supplemented with rifampicin at 0.5 µg/mL. After outgrowth, colonies were picked into 7H9 media. After 1 week of outgrowth, an aliquot was heat inactivated and the rifampicin resistance determining region (RRDR) of *rpoB*, *rpoA*, and *rpoC* were PCR-amplified and Sanger sequenced. See **Supplemental Table 4** for primer sequences.

Generation of structural models

The structural model of Mtb RNAP transcription initiation complex (TIC) bound to Rif in **Figure 1A** was generated by modeling *Mycobacterium smegmatis* RNAP bound to Rif (PDB 6CCV[65]) on to the TIC structure (PDB 6EDT[66]).

The cryo-EM structures of a NusG-bound paused elongation complex from Mtb (PDB 8E74) in **Figure 2D,** and the location of clinical isolate mutations in **Figure 4A** are derived from Delbeau et al.[13]

Generation of individual CRISPRi strains

Individual CRISPRi plasmids were cloned as described in Wong and Rock[67] using Addgene plasmid 166886. Briefly, the CRISPRi plasmid backbone was digested with BsmBI-v2 (NEB R0739L) and gel purified. sgRNAs were designed to target the non-template strand of the target gene ORF. For each individual sgRNA, two complementary oligonucleotides with appropriate sticky end overhangs were annealed and ligated (T4 ligase NEB M0202M) into the BsmBI-digested plasmid backbone. Successful cloning was confirmed by Sanger sequencing.

Individual CRISPRi plasmids were then electroporated into Mtb. Electrocompetent cells were obtained as described in [68]. Briefly, an Mtb culture was expanded to an OD600 = 0.4–0.6 and treated with glycine (final concentration 0.2M) for 24 h before pelleting (4,000 × g for 10 min). The cell pellet was washed three times in sterile 10% glycerol. The washed bacilli were then resuspended in 10% glycerol in a final volume of 5% of the original culture volume. For each transformation, 100 ng plasmid DNA and 100 µl electrocompetent mycobacteria were mixed and transferred to a 2 mm electroporation cuvette (Bio-Rad 1652082). Where necessary, 100 ng plasmid pIRL19 (Addgene plasmid 163634) was also added. Electroporation was performed using

the Gene Pulser X cell electroporation system (Bio-Rad 1652660) set at 2,500 V, 700 Ω and 25 µF. Bacteria were recovered in 7H9 for 24 h. After the recovery incubation, cells were plated on 7H10 agar supplemented with the appropriate antibiotic to select for transformants.

CRISPRi library transformation
CRISPRi libraries were generated as described in Bosch et al.[28] Briefly, fifty transformations were performed to generate RifS and βS450L Δ*bioA* libraries. For each transformation, 1 µg of RLC12 plasmid DNA was added to 100 µL electrocompetent cells. The cells:DNA mix was transferred to a 2 mm electroporation cuvette (Bio-Rad #1652082) and electroporated at 2500 kV, 700 ohms, and 25 µF. Each transformation was recovered in 2 mL 7H9 media supplemented with OADC, glycerol and Tween80 (100 mL total) for 16-24 hours. The recovered cells were harvested at 4,000 rpm for 10 minutes, resuspended in 400 µL remaining media per transformation and plated on 7H10 agar supplemented with kanamycin (see Mycobacterial cultures) in Corning Bioassay dishes (Sigma #CLS431111-16EA).

After 21 days of outgrowth on plates, transformants were scraped and pooled. Scraped cells were homogenized by two dissociation cycles on a gentleMACS Octo Dissociator (Miltenyi Biotec #130095937) using the RNA_01 program and 30 gentleMACS M tubes (Miltenyi Biotec #130093236). The library was further declumped by passaging 1 mL of homogenized library into 100 mL of 7H9 supplemented with kanamycin (see Mycobacterial cultures) for between 5 and 10 generations. Final RifS and βS450L Δ*bioA* Mtb library stocks were obtained after passing the cultures through a 10 µm cell strainer (Pluriselect #SKU 43-50010-03). Genomic DNA was extracted from the final stocks and library quality was validated by deep sequencing (see Genomic DNA extraction and library preparation for Illumina sequencing).

Pooled CRISPRi screen
Pooled CRISPRi screens were performed as described in Bosch et al[28]. Briefly, twenty mL cultures were grown in vented tissue culture flasks (T-75; Falcon #353136) and 7H9 media supplemented with kanamycin (see Mycobacterial cultures) and maintained at 37°C, 5% CO2 in a humidified incubator.

The screen was initiated by thawing four 1 mL aliquots of the *M. tuberculosis* Δ*bioA* (RifS or βS450L) CRISPRi library (RLC12) and inoculating each aliquot into 24 mL 7H9 media supplemented with kanamycin in a T-75 flask (starting OD_{600}~0.06). The cultures were expanded to approximately OD_{600} = 1.0, pooled and passed through a 10 µm cell strainer (pluriSelect #43-50010-03) to obtain a single cell suspension. The single cell suspension (flow-though) was used to set up six “generation 0” cultures: three replicate cultures with ATc (+ATc) and three replicate control cultures without ATc (–ATc). From each generation 0 culture, we harvested 10 OD_{600} units of bacteria (~$3x10^9$ bacteria; ~30,000X coverage of the CRISPRi library) for genomic DNA extraction. The remaining culture volume was used to initiate the pooled CRISPRi fitness screen. Cultures were periodically passaged in pre-warmed media in order to maintain log phase growth. At generation 2.5, 5, and 7.5, cultures were back-diluted 1:6 (to a starting OD_{600} = 0.2) and cultivated for approximately 2.5 doublings. At generation 10, 15, 20, and 25, cultures were back-diluted 1:24 (to a starting OD_{600} = 0.05) and expanded for 5 generations before reaching late-log phase. ATc was replenished at every passage. By keeping the OD_{600} of the 20 mL cultures ≥ 0.05, we guaranteed sufficient coverage of the library (3,000X) at all times. At set time points (approximately 2.5; 5; 7.5; 10; 15; 20; 25 and 30 generations), we harvested bacterial pellets (10 OD_{600} units) to extract genomic DNA.

Genomic DNA extraction and library preparation for Illumina sequencing of CRISPRi libraries

Genomic DNA was isolated from bacterial pellets using the CTAB-lysozyme method described by Larsen et al [69]. Genomic DNA concentration was quantified using the DeNovix dsDNA high sensitivity assay (KIT-DSDNA-HIGH-2; DS-11 Series Spectrophotometer / Fluorometer).

Illumina libraries were constructed as described in Bosch et al[28]. Briefly, the sgRNA-encoding region was amplified from 500 ng genomic DNA using NEBNext Ultra II Q5 master Mix (NEB #M0544L). PCR cycling conditions were: 98°C for 45 s; 17 cycles of 98°C for 10 s, 64°C for 30 s, 65°C for 20 s; 65°C for 5 min. Each PCR reaction a unique indexed forward primer (0.5 μM final concentration) and a unique indexed reverse primer (0.5 μM) (**Supplemental Table 4**). Forward primers contain a P5 flow cell attachment sequence, a standard Read1 Illumina sequencing primer binding site, custom stagger sequences to ensure base diversity during Illumina sequencing, and unique barcodes to allow for sample pooling during deep sequencing. Reverse primers contain a P7 flow cell attachment sequence, a standard Read2 Illumina sequencing primer binding site, and unique barcodes.

Following PCR amplification, each ~230 bp amplicon was purified using AMPure XP beads (Beckman–Coulter #A63882) using two-sided selection (0.75X and 0.12X). Eluted amplicons were quantified with a Qubit 2.0 Fluorometer (Invitrogen), and amplicon size and purity were quality controlled by visualization on an Agilent 4200 TapeStation (Instrument- Agilent Technologies #G2991AA; reagents- Agilent Technologies #5067-5583; tape- Agilent Technologies #5067-5582). Next, individual PCR amplicons were multiplexed into 20 nM pools and sequenced on an Illumina sequencer according to the manufacturer's instructions. To increase sequencing diversity, a PhiX spike-in of 2.5–5% was added to the pools (PhiX sequencing control v3; Illumina FC-110-3001). Samples were run on the Illumina NextSeq 500 or NovaSeq 6000 platform (single-read 1 ×85 cycles, 8 × i5 index cycles, and 8 × i7 index cycles).

Differential vulnerability analysis of Rif Resistant versus Rif Sensitive Strains

Gene vulnerability in the RifS and βS450L *M. tuberculosis* strains was determined using an updated vulnerability model based on the one previously described[28]. In the updated model, read counts for a given sgRNA in the minus ATc conditions were modeled using a Negative Binomial distribution with a mean proportional to the counts in the plus ATc condition, plus a factor representing the log2 fold-change:

$$y_i^{-ATc} \sim NegBinom(\eta_i, \phi)$$

$$\eta_i = \log\left(y_i^{+ATc} + \lambda_i\right) + TwoLine(x_i, \alpha_l, \beta_l, \gamma, \beta_e)$$

where λ_i is an sgRNA-level correction factor estimated by the model, x_i represents the generations analyzed for the i-th guide, and the TwoLine function represents the piecewise linear function previously described, which models sgRNA behavior over he logistic function describing gene-level vulnerabilities was simplified by setting the top asymptote of the curve (previously "K") equal to 0, representing the fact that weakest possible sgRNAs are expected to impose no effect on bacterial fitness i.e.:

$$Logistic(s) = \frac{\beta_{max}}{\left(1 + e^{(-H\cdot(s-M))}\right)}$$

The Bayesian vulnerability model was run for each condition independently, and samples for all the parameters were obtained using Stan running 4 independent chains with 1,000 warmup iterations and 3000 samples each (for a total of 12,000 posterior samples for each parameter in the model after discarding warmup iterations).

Differential vulnerabilities were estimated by two approaches. First, for each gene, the difference in pairwise (guide-level) vulnerability estimates was obtained, resulting in posterior samples of the differential vulnerability (delta-vulnerability). This effectively estimated the difference in the integrals of the vulnerability functions. If the 95% credible region did not overlap 0.0 those were taken as significant differential vulnerabilities between the strains.

Next, to identify differences between genes which may not exhibit the expected dose-response curve, we estimated the fitness cost (log2FC) predicted by our model for a (theoretical) sgRNA of strength 0.0 (i.e. $Logistic(s = 0)$). This represented the weakest phenotype theoretically possible with our CRISPRi system, which we call "F_{min}". The difference between this value was estimated for each gene (ΔF_{min}) and those where the 95% credible region did not overlap 0.0 were identified as significant differential vulnerabilities by this approach.

Pathway analysis

First, all annotated Mtb genes were associated with a pathway as defined by the Kyoto Encyclopedia of Genes and Genomes (KEGG) database[70–72]. If necessary, annotations were manually-curated to update or correct pathway assignments. To quantify pathway enrichment, the query set was defined as the union of the upper quartile of differential vulnerabilities defined by both the original gene vulnerability calling method (ΔV) and the F_{min} approach. The background set was defined as all annotated Mtb genes. Enrichment of the pathways identified as differentially vulnerable was calculated by an odds ratio and significance was determined with a Fisher's exact test.

phyOverlap

To detect associations between gene variants and rifampicin-resistance, we employed a phylogenetic convergence test using the *phyOverlap* algorithm[73] (https://github.com/Nathan-d-hicks/phyOverlap). Briefly, FASTQ files were aligned to H37Rv genome (NC_018143.2) using bwa (version 0.7.17-r1188). FASTQ accession numbers are provided in **Supplemental Table 3**. SNPs were called and annotated using the HaplotypeCaller tool Genome Analysis Toolkit (version 3.5) using inputs from samtools (version 1.7). SNP sites with less than 10x coverage or missing data in >10% strains were removed from the analysis. Repetitive regions of the genome (PE/PPE genes, transposases, and prophage genes) are excluded from the analysis. Known drug-resistance (DR) regions were further excluded so as not to bias phylogenetic tree construction. *M. canetti* was provided as an outgroup (NC_015848). We performed Maximum Likelihood Inference using RAxML (v8.2.11) to construct the ancestral sequence and determine the derived state of each allele. Overlap with rifampicin-resistance was scored by dividing the number of genotypically-predicted (Mykrobe v0.9.012) RifR isolates containing a derived allele by the total number of isolates with a derived allele at a given genomic position. To generate a gene-wide score, we excluded synonymous SNPs and averaged the individual nonsynonymous SNP scores, weighting the scores by the number of times derived alleles evolved across the phylogenetic tree. The significance of the overlap is then tested by redistributing mutation events for each SNP randomly across the tree and recalculating the score. This permutation is done 50,000 times to derive the p-value. This analysis additionally used FastTree (version 2.1.11) and figTree (v1.4.4).

dN/dS calculations

The ratio of nonsynonymous (dN) to synonymous (dS) nucleotide substitutions was used to quantify selective pressure acting on *nusG* and *rpoC*. A dN/dS value less than one suggests negative or purifying selection whereas a dN/dS value greater than one suggests positive or diversifying selection. For this analysis, we used a collection of ~50,000 Mtb clinical isolate whole genome sequences, as described in Li et al[41]. Isolates were grouped based on the presence of genotypically-predicted rifampicin-resistance (Mykrobe v0.9.012), as well as the identity of the *rpoB* mutation (S450X or H445X; where X indicates any amino acid other than S or H, respectively) conferring RifR. The number of samples used in the *nusG* dN/dS analysis shown in Figure 3 are as follows: 1,365 RifS, 350 RifR, 270 S450X, and 26 H445X. The number of samples used in the *rpoC* dN/dS analysis shown in Figure 3 are as follows: 23,024 RifS, 13,993 RifR, 11,067 S450X, and 1,215 H445X. Insertions and deletions were necessarily excluded from this analysis. A bootstrap-analysis was performed to calculate the dN/dS ratios to reduce any potential effects of recent clonal expansion events or convergent evolution of a specific site, like acquired drug resistance mutations, as performed in Liu et al[42]. The analysis was performed by sub-sampling 80% of total variants in each group. The sub-sampling was repeated 100 times. dN/dS values were calculated for each subset of samples using a python script obtained from the github repository: https://github.com/MtbEvolution/resR_Project/tree/main/dNdS.

SNP calling and Upset Plot

SNP information for all Mtb clinical isolate whole genome sequences were called as follows. FASTQ reads were aligned to the H37Rv genome (NC_018143.2) and SNPs were called and annotated using Snippy9 (version 3.2-dev) using default parameters (minimum mapping quality of 60 in BWA, samtools base quality threshold of 20, minimum coverage of 10, minimum proportion of reads that differ from reference of 0.9). Mapping quality and coverage was further assessed using QualiMap with the default parameters (version 2.2.2-dev). Samples with a mean coverage < 30, mean mapping quality <= 45, or GC content <= 50% or >= 70% were excluded. Drug resistance conferring SNPs were annotated using Mykrobe (v0.9.012). The resulting SNP and drug-resistance calls were used to generate the values depicted in the upset plot.

Phylogenetic Trees

Phylogenetic trees based on SNP calls described above were built using FastTree (version 2.1.11 SSE3). A list of SNPs in essential genes was concatenated to build phylogenetic trees. Indels, drug resistance-conferring SNPs, and SNPs in repetitive regions of the genome (PE/PPE genes, transposases and prophage genes) were excluded. Tree visualization was performed in iTol (https://itol.embl.de/).

Barcode (BC) library production

The barcode library was designed to include over 100,000 random 18-mer sequences cloned into an Giles-integrating backbone (*attP* only, no Integrase) containing a hygromycin resistance cassette with a premature stop codon (pINP472). Oligonucleotides were synthesized as a gBlocks Library by IDT, containing 104,976 fragments.

1.6 µg of pINP472 was digested with PciI (NEB #R0655) and gel purified (QIAGEN #28706). The library was PCR amplified using NEBNext High-Fidelity 2X PCR Master Mix (NEB #M0541L). One 50 µL reaction was prepared, containing 25 µL of PCR master mix, 0.0125 pmol of the gBlock library, and a final concentration of 0.5 µM of the appropriate forward and reverse primers (Fwd: 5'-TTACGCGTTTCACTGGCCGATTG-3' + Rv: 5'-TTTTGCTGGCCTTTTGCTCAAC-3'). PCR cycling conditions were: 98°C for 30 s; 15 cycles of 98°C for 10 s, 68°C for 10 s, 72°C for 15 s; 72°C for 120 s. The PCR amplicon were purified using the QIAGEN MinElute PCR purification kit (QIAGEN #28004). One Gibson assembly reaction (NEB #E2621) was prepared with 0.01

pmol/µL digested pINP472 backbone, 0.009 pmol/µL cleaned PCR amplicon, and master mix, representing a 1:2 molar ratio of vector:insert.

Following incubation at 50°C for 1 h, 7 µL the Gibson product was dialyzed to remove salts and transformed into 100 µL MegaX DH10B T1R Electrocomp Cells (Invitrogen #C640003) diluted with 107 µL 10% glyerol. For each of 3 total transformations, 75 µL of the cells:DNA mix was transferred to a 0.1 cm electroporation cuvette (BioRad #1652089) and electroporated at 2,000 V, 200 ohms, 25 µF. Transformations were washed twice with 300 µL provided recovery media and recovered in a total of 3 mL media. Cells were allowed to recover at 37°C with gentle rotation. Recovered cells were plated across three plates of LB agar supplemented with zeocin. After 1 d incubation at 37°C, transformants were scraped and pooled. One fourth of the pellet (3.2g dry mass) was used to perform 24 minipreps using a QIA prep Spin Miniprep Kit (Qiagen #27104).

Transformation of BC library into Mtb

The barcode library was transformed into RifS and βS450L Mtb expressing RecT (mycobacteriophage recombinase) similarly to the CRISPRi library (see CRISPRi library transformation), with minor modifications. Briefly, cultures for competent cells were grown in 7H9 supplemented with kanamycin to retain the episomal *recT* encoding plasmid (pIRL4). 20 mL cultures were concentrated ten times and transformed with 250 ng of library and 100 ng of non-replicating, Giles integrase containing plasmid (pIRL40). Additionally, after recovery cells were plated on 7H10 agar supplemented with kanamycin and zeocin. Transformants were scrapped after 29 days of outgrowth.

ssDNA recombineering & validation of strains

Clinical *nusG*, *rpoB*, and *rpoC* mutants were introduced into RifS and βS450L Mtb using oligo-mediated (single stranded DNA) recombineering, as described by Murphy et al[68]. Briefly, 70-mer oligos were designed to correspond to the lagging strand of the replication fork, with the desired mutation in the middle of the sequence. Alterations were chosen to avoid recognition by the mismatch-repair machinery of RecT expression was induced ~16 h before transformation by addition of ATc to a final concentration of 0.5 µg/mL. 400 µL of competent cells were transformed with 5 µg of mutation containing oligo and 0.1 µg of hygromycin resistance cassette repair oligo (1:50 ratio of mutant oligo to repair oligo) and recovered in 5 mL 7H9 media.

After 24 hours of recovery, 200 µL of cells were plated on 7H10 plates supplemented with hygromycin. After 21 days of outgrowth, 12 colonies per construct were picked into 100 µL 7H9 media supplemented with hygromycin in a 96 well plate (Fischer Scientific #877217). 50 µL of culture were heat inactivated at 80°C for 2 h in a sealed microamp 96 well plate (Fischer Scientific #07200684; Applied Biosystems #N8010560). 50 µL of heat inactivated culture was mixed with 50 µL of 25% DMSO and lysed at 98°C 10 minutes.

Mutations of interest and unique barcodes were confirmed with PCR amplification and Sanger sequencing. The region of interest was PCR amplified with NEBNext High-Fidelity 2X PCR Master Mix (NEB #M0541L) using 0.5 µL of heat lysed product with the appropriate primers, annealing temperatures, and extension times (see **Supplemental Table 4**). Residual PCR primers were removed with NEB Shrimp Alkaline Phosphatase (rSAP) and exonuclease I (exo) (rSAP- NEB # M0371; exo- NEB # M0293) per manufacturer's instructions. Amplicons were then submitted for Sanger sequencing. One to three unique independent isolates were generated for all tested mutations.

Pooled BC competitive growth assay

Validated mutants were first grown in 1mL 7H9 with hygromycin and after 3 days, expanded to 5 mL 7H9 with hygromycin. Strains were pooled to contain approximately 1.2 x 10^7 cells for each mutant. The pool was then diluted to a starting OD600 of 0.01 in 7H9 supplemented with hygromycin. At this point, three 20 mL cultures in vented tissue culture flasks (T-75; Falcon #353136) were expanded to late log phase and used as input for the competitive growth experiment. 16 OD600 units of cells were harvested from flask as the input culture (generation 0). Triplicate cultures were then diluted back to OD600 = 0.05 and grown for ~4.5 generations, back-diluted again to OD600 = 0.05 and grown for an additional 4 generations. After this, cultures were harvested for a cumulative 8.5 generations of competitive growth.

Genomic DNA extraction and library preparation for next-generation sequencing followed the same protocol as that of the CRISPRi libraries (see above), with minor modifications. Briefly, the barcode region was amplified from 100 ng genomic DNA using NEBNext Ultra II Q5 master Mix (NEB #M0544L). PCR cycling conditions were: 98°C for 45 s; 16 cycles of 98°C for 10 s, 64°C for 30 s, 65°C for 20 s; 65°C for 5 min. Each PCR reaction contained a unique indexed forward primer (0.5 μM final concentration) and a unique indexed reverse primer (0.5 μM) (see **Supplemental Table 4**). Additionally, individual PCR amplicons were multiplexed into a 1 nM pool and sequenced on an Illumina sequencer according to the manufacturer's instructions. To increase sequencing diversity, a PhiX spike-in of 20% was added to the pool (PhiX sequencing control v3; Illumina FC-110-3001). Samples were run on the Illumina MiSeq Nano platform (paired-read 2 ×150 cycles, 8 × i5 index cycles, and 8 × i7 index cycles).

WGS and SNP calling for passaging timepoints and ssDNA recombinants

Genomic DNA (gDNA) was extracted as described above. gDNA was diluted and subjected to Illumina whole genome sequencing by SeqCenter. Briefly, Illumina libraries were generated through tagmentation-based and PCR-based Illumina DNA Prep kit and custom IDT 10bp unique dial indices, generating 320 bp amplicons. Resulting libraries were sequenced on the Illumina NovaSeq 6000 platform (2 ×150 cycles). Demultiplexing quality control, and adapter trimming was performed with bcl-convert (v4.1.5).

Reads were aligned to the *M. tuberculosis* (H37Rv; CP003248.2) reference genome using bwa (v1.3.1) with default parameters. Variant detection was performed by Snippy (v4.6.0)/freebayes (v1.3.1). Resulting vcf files were inspected for compensatory mutations (**Supplemental Table 2**) in *rpoABC* and/or the presence of the desired mutation.

Definition of putative compensatory *nusG, rpoA, rpoB, rpoC* variants

Compensatory mutations in *rpoA*, *rpoB*, and *rpoC* were taken from published sources and are described in **Supplemental Table 2**. Inclusion as a putative compensatory mutation in our list required that each reported variant in *rpoA*, *rpoB*, or *rpoC* was found specifically in rifampicin-resistant strains, defined here as meaning that ≥90% of all strains harboring the putative compensatory mutation were genotypically-predicted (gDST) RifR. The use of the ≥90% gDST RifR cutoff allows for presumptive instances of incorrect gDST calls for strains harboring rare compensatory variants. The strains used for this analysis are the approximately 50,000 Mtb WGS strain collection described in Li et al[41].

The rules to define putative compensatory *nusG* mutations are as follows. Each *nusG* variant observed was assessed according to the following three rules and, if it met one of them, was deemed a putative compensatory variant.

1) The *nusG* variant was found in ≥80% genotypically-predicted (gDST) RifR strains and was present in at least two distinct Mtb (sub)lineages. The use of the ≥80% gDST RifR cutoff

allows for presumptive instances of incorrect gDST calls for strains harboring rare *nusG* variants.

2) The *nusG* variant was found in 100% gDST RifR strains but only present in a single Mtb sublineage, but the same or nearby NusG site (+/– 5 amino acids) was also mutated to an alternative amino acid that met the criteria stated in rule 1.
3) Residues based on the Mtb NusG-RNAP structure[13] that were predicted to be important for the NusG pro-pausing activity (e.g. NusG Trp120).

The rules to define a putative compensatory mutation in the *rpoB* β protrusion were similar to those described for *nusG*, except that only *rpoB* β protrusion residues at or near the NusG interface (RpoB Arg392-Thr410) were included in the analysis. Note that two such β protrusion mutations (Thr400Ala and Gln409Arg) were previously identified as putative compensatory mutation[17,74,75] (**Supplemental Table 2**).

RifR *rpoB* allele frequency distribution calculations

To check whether the observed distribution of RifR *rpoB* mutations was different for each of the three groups (all RifR strains in our clinical strain genome database, those harboring known compensatory mutations in *rpoA* or *rpoC*, or those harboring compensatory mutations in *nusG* or the β protrusion), we performed a chi-squared test on the observed RifR *rpoB* mutant frequencies. Specifically, we take the RifR *rpoB* mutant frequencies observed in all RifR samples as representing an estimate of the base probabilities under the null hypothesis. We then use these base probabilities to calculate the frequency of mutations that would be expected in the other groups, based on the null-hypothesis. That is:

For each mutation (m):

$$p(m) = \frac{\text{Number of times } m \text{ occurs in RifR samples}}{\text{Total number of RifR samples}}$$

For each group (G) and mutation (m),

$$E[m|G] = p(m) \times \text{Total number of samples in } G$$

Protein expression and purification

Mtb RNAP

Mtb RNAP was purified as previously described[66,76]. Briefly, plasmid pMP61 (WT RNAP) or pMP62 (S450L RNAP) was used to overexpress *Mtb* core RNAP subunits *rpoA*, *rpoZ*, a linked *rpoBC* and a His_8-tag. pMP61/pMP62 was grown in *Eco* Rosetta2 cells in LB with 50 µg kanamycin/mL and 34 µg chloramphenicol/mL at 37 °C to an OD_{600} of 0.3, transferred to room temperature and left shaking to an approximate OD_{600} of 0.6. RNAP expression was induced by adding IPTG to a final concentration of 0.1 mM, grown for 16 hr, and harvested by centrifugation (8000 x g, 15 min at 4 °C). Harvested cells were resuspended in 50 mM Tris-HCl, pH 8.0, 1 mM EDTA, 1 mM PMSF, 1 mM protease inhibitor cocktail, 5% glycerol and lysed by sonication. The lysate was centrifuged (27,000 x *g*, 15 min, 4 °C) and polyethyleneimine (PEI, Sigma-Aldrich) added to the supernatant to a final concentration of 0.6% (w/v) and stirred for 10 min to precipitate DNA binding proteins including target RNAP. After centrifugation (11000 x *g*, 15 min, 4 °C), the pellet was resuspended in PEI wash buffer (10 mM Tris-HCl, pH 7.9, 5% v/v glycerol, 0.1 mM EDTA, 5 mM DTT, 300 mM NaCl) to remove non-target proteins. The mixture was centrifuged (11000 x g, 15 min, 4 °C), supernatant discarded, then RNAP eluted from the pellet into PEI Elution Buffer (10 mM Tris-HCl, pH 7.9, 5% v/v glycerol, 0.1 mM EDTA, 5 mM DTT, 1 M NaCl). After centrifugation, RNAP was precipitated from the supernatant by adding $(NH_4)_2SO_4$ to a final

concentration of 0.35 g/L. The pellet was dissolved in Nickel buffer A (20 mM Tris pH 8.0, 5% glycerol, 1 M NaCl, 10mM imidazole) and loaded onto a HisTrap FF 5 mL column (GE Healthcare Life Sciences). The column was washed with Nickel buffer A and then RNAP was eluted with Nickel elution buffer (20 mM Tris, pH 8.0, 5% glycerol, 1 M NaCl, 250 mM imidazole). Eluted RNAP was subsequently purified by gel filtration chromatography on a HiLoad Superdex 26/600 200 pg in 10mM Tris pH 8.0, 5% glycerol, 0.1mM EDTA, 500mM NaCl, 5mM DTT. Eluted samples were aliquoted, flash frozen in liquid nitrogen and stored in -80 °C until usage.

Mtb σ^A–RbpA

Mtb σA–RbpA was purified as previously described[76,77]. The Mtb σA expression vector pAC2 contains the T7 promoter, ten histidine residues, and a precision protease cleavage site upstream of Mtb σA. The Mtb RbpA vector is derived from the pET-20B backbone (Novagen) and contains the T7 promoter upstream of untagged Mtb RbpA. Both plasmids were co-transformed into Eco Rosetta2 cells and selected on media containing Kanamycin (50 µg/mL), Chloramphenicol (34 µg/mL) and Ampicillin (100 µg/mL). Protein expression was induced at OD600 of 0.6 by adding IPTG to a final concentration of 0.5 mM and leaving cells to grow at 30 °C for 4 hours. Cells were then harvested by centrifugation (4,000 x g, 20 min at 4 °C). Harvested cells were resuspended in 50 mM Tris-HCl, pH 8.0, 500 mM NaCl, 5mM imidazole, 0.1 mM PMSF, 1 mM protease inhibitor cocktail, and 1 mM ß-mercaptoethanol, then lysed using a continuous- flow French press. The lysate was centrifuged twice (15,000 x g, 30 min, 4 °C) and the proteins were purified by Ni2+-affinity chromatography (HisTrap IMAC HP, GE Healthcare Life Sciences, Pittsburgh, PA) via elution at 50 mM Tris-HCl, pH 8.0, 500 mM NaCl, 500 mM imidazole, and 1 mM ß-mercaptoethanol. Following elution, the complex was dialyzed overnight into 50mM Tris-HCl, pH 8.0, 500 mM NaCl, 5 mM imidazole, and 1 mM ß-mercaptoethanol and the His10 tag was cleaved with precision protease overnight at a ratio of 1/30 (protease mass/cleavage target mass). The cleaved complex was loaded onto a second Ni2+-affinity column and was retrieved from the flow-through. The complex was loaded directly onto a size exclusion column (SuperDex-200 16/16, GE Healthcare Life Sciences) equilibrated with 50 mM Tris-HCl, pH 8, 500mM NaCl, and 1mM DTT. The sample was concentrated to 4 mg/mL by centrifugal filtration and stored at –80 °C until usage.

Mtb CarD

Mtb CarD was purified as previously described[66,76]. In brief, Mtb CarD was overexpressed from pET SUMO (Invitrogen) in Eco BL21(DE3) cells (Novagen) and selected on media containing 50 µg/mL Kanamycin. Protein expression was induced by adding IPTG to a final concentration of 1 mM when cells reached an apparent OD_{600} of 0.6, followed by 4 hours of growth at 28 °C, then harvested by centrifugation (4,000 x g, 15 min at 4 °C). Harvested cells were resuspended in 20 mM Tris-HCl, pH 8.0, 150 mM K-Glutamate, 5mM $MgCl_2$, 0.1 mM PMSF, 1 mM protease inhibitor cocktail, and 1 mM ß-mercaptoethanol, then lysed using a continuous-flow French press. The lysate was centrifuged twice (16,000 x g, 30 min, 4 °C) and the proteins were purified by Ni^{2+}-affinity chromatography (HisTrap IMAC HP, GE Healthcare Life Sciences, Pittsburgh, PA) via elution at 20 mM Tris-HCl, pH 8.0, 150 mM K-Glutamate, 250 mM imidazole, and 1 mM ß-mercaptoethanol. Following elution, the complex was dialyzed overnight into 20 mM Tris-HCl, pH 8.0, 150 mM K-Glutamate, 5 mM $MgCl_2$, and 1 mM ß-mercaptoethanol and the His_{10} tag was cleaved with ULP-1 protease (Invitrogen) overnight at a ratio of 1/30 (protease mass/ cleavage target mass). The cleaved complex was loaded onto a second Ni^{2+}-affinity column and was retrieved from the flow-through. The complex was loaded directly onto a size exclusion column (SuperDex-200 16/16, GE Healthcare Life Sciences) equilibrated with 20 mM Tris-HCl, pH 8, 150 mM K-Glutamate, 5 mM $MgCl_2$ and 2.5mM DTT. The sample was concentrated to 5mg/mL by centrifugal filtration and stored at –80 °C.

WT Mtb NusG (+ mutants N65H, R124L, N125S)
Plasmid pAC82 (or mutant variation) was used to overexpress WT Mtb NusG[13]. Plasmids encoding NusG mutants were generated using Q5 Site-directed mutagenesis (NEB) and sequenced to confirm the presence of target mutations. *Eco* BL21 cells containing plasmids encoding different versions of *Mtb* NusG were grown in LB with 50 µg kanamycin/mL at 37 °C to an OD_{600} of 0.4, then transferred to room temperature and left shaking to an OD_{600} of 0.67. Protein expression was induced by adding IPTG to a final concentration of 0.1 mM, grown for an additional 4 hr, then harvested by centrifugation (4,000 x *g*, 20 min at 4 °C). Harvested cells were resuspended in 50 mM Tris-HCl, pH 8.0, 500 mM NaCl, 5 mM imidazole, 10% glycerol, 1 mM PMSF, 1 mM protease inhibitor cocktail (Roche), 2 mM β-mercaptoethanol, and lysed by French press. The lysate was centrifuged (4,000 rpm for 20 min, 4 °C) and the supernatant was removed and applied to a HisTrap column pre-washed with 50 mM Tris-HCl, pH 8.0, 500 mM NaCl, 10% glycerol, 15 mM imidazole, and 2 mM β-mercaptoethanol. After loading the sample, the column was washed with five volumes of the same buffer, before gradient elution with 50 mM Tris-HCl, pH 8.0, 500 mM NaCl, 10% glycerol, 250 mM imidazole, and 2 mM β-mercaptoethanol. The eluted protein was mixed with precision protease and dialyzed overnight at 4 °C in 20 mM Tris-HCl, pH 8.0, 500 mM NaCl, 10 mM β-mercaptoethanol to cleave the N-terminal His10-tag before applying to a HisTrap column to remove the uncleaved protein. The flow-through was collected and glycerol was added to a final concentration of 20% (v/v). Aliquots were flash frozen in liquid nitrogen and stored in –80 °C until use.

Promoter-based *in vitro* termination assays
The DNA sequence for the Mycobacterium tuberculosis H37Rv 5S rRNA (*rrf* gene) intrinsic terminator was taken from Mycobrowser (MTB000021), with genomic coordinates of 1,476,999 to 1,477,077 basepairs. The intrinsic terminator was found by predicting its RNA structure using mfold (RNA folding form v2.3) via the UNAFold Web Server. The intrinsic terminator was cloned downstream of a cytidine-less halt cassette in plasmid pAC70[38], a gift of the Robert Landick lab (University of Wisconsin-Madison), using Q5 site-directed mutagenesis (following manufacturer's protocol – NEB) at an annealing temperature of 59°C with GC enhancer for the PCR step, with primers 5'-TGGTGTTTTTGTATGTTTATATCGACTCAGCCGCTCGCGCCATGGACGCTCTCCTGA-3' and 5'-CCGTTACCGGGGGTGTTTTTGTATGTTCGGCGGTGTCCTGGATCCTGGCAGTTCCCT-3' (synthesized by IDT), to create plasmid pJC1. The 323 base pairs linear DNA fragment used for in vitro transcription assays was PCR amplified using Accuprime Pfx DNA polymerase (Invitrogen™) at an annealing temperature of 56.5°C, with primers 5'-GAATTCAAATATTTGTTGTTAACTCTTGACAAAAGTGTTAAAAGC-3' and 5'-GTTGCTTCGCAACGTTCAAATCC-3' (synthesized by IDT), following manufacturer instructions, and PCR purified (using the QIAquick® PCR Purification Kit – QIAGEN®) to remove protein contents and buffer exchange into 10mM Tris-HCl pH 8.5.

pJC1 contains the *rrf* termination site at approximately +150bp. This template also contained a C-less cassette (+1 to +26). Core RNAP was incubated for 15 min at 37 °C with σA/RbpA in transcription buffer (20 mM Tris, 25 mM KGlu, 10 mM MgOAc, 1 mM DTT, 5 µg/mL BSA) to form holo-RNAP, followed by 10 min incubation with 500 nM CarD at 37 °C. Holo-RNAP (200 nM) was then incubated with template DNA (10 nM) for 15 min at 37 °C. To initiate transcription, the complex was incubated with ATP + GTP (both at 16 µM), UTP (2 µM), and 0.1 µL/reaction [α-^{32}P]UTP for 15 min at 37 °C to form a halted complex at U26. Transcription was restarted by adding a master mix containing NTP mix (A + C + G + U), heparin, and NusG at a final concentration of 150 µM (each NTP), 10

μg/mL (heparin), and 1 μM NusG at 23 °C. The reaction was allowed to proceed for 30 min, followed by a “chase” reaction in which all 4 nucleotides were added to a final concentration of 500 μM each. After 10 min, aliquots were removed and added to a 2X Stop buffer (95% formamide, 20 mM EDTA, 0.05% bromophenol blue, 0.05% xylene cyanol). Samples were analyzed on an 8% denaturing PAGE (19:1 acrylamide: bis acrylamide, 7M urea, 1X TBE pH= 8.3) for 1.25 hr at 400 V, and the gel was exposed on a Storage Phosphor Screen and imaged using a Typhoon PhosphoImager (GE Healthcare).

Quantitation of termination and changes in termination

Synthesized RNA bands on the gel image were quantified using Image J software (NIH). Each lane from below the *rrf* termination site (~150 nt) to above the run-off RNA products (263 nt) was converted to a pseudo-densitometer plot using the Image J line function and the relative areas of the termination and run-off bands were measured. Termination efficiency (TE) was calculated as the fraction of the termination (term) peak area relative to total of the termination and run-off (term + runoff) peak areas. Fold changes in termination attributable to each NusG (ΔT) were determined as the aggregate of changes in the termination rates k_b and k_t, as defined by von Hippel and Yager [equations (1) and (2)][61,62]. Multiple algebraic transforms can yield the aggregate fold-changes in termination, ΔT, based on the following equations.

(1)

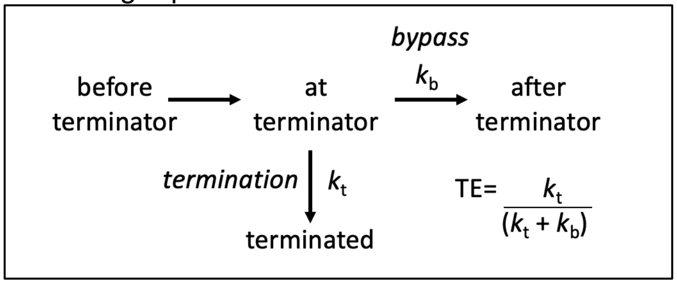

(2) $$TE = [1 + e^{-\Delta\Delta G^{\ddagger} / -RT}]^{-1},$$

where $\Delta\Delta G^{\ddagger}$ is the difference in activation barriers between termination and bypass, which is most directly related to the energies of RNAP–NusG and internal RNAP interactions that govern termination.

(3) $$\Delta\Delta G^{\ddagger} = -RT*\ln((1/TE)-1)$$

(equation (2) rearranged)

(4) $$\Delta T = e^{(\Delta\Delta G^{\ddagger}2-\Delta\Delta G^{\ddagger}1)}$$

(fold change in aggregate termination rates for two conditions, 1 and 2.)

(5) $$\Delta T = \frac{\left(\frac{1}{TE_2}\right)-1}{\left(\frac{1}{TE_1}\right)-1}$$

(alternative calculation derived from equation (1) assuming NusG only affects k_b)

Calculating ΔT using either the combinations of equations (3) and (4) or using equation (5) gives the same results because the ΔT is the same whether conditions differ by aggregate effects on both k_b and k_t or an effect on only one of them. We calculate ΔT using these approaches rather than the simple difference in energies of activation ($\Delta\Delta G^{\ddagger}2-\Delta\Delta G^{\ddagger}1$) because it allows a clearer graphical depiction of effects without changing the results. Errors in ΔT were calculated using a two-sided, unpaired t-test with no assumptions on variance.

Electrophoretic Mobility Shift Assay (EMSA)

RNAP-NusG complexes were assembled and run on an EMSA to test proper binding of all mutant NusGs. Core RNAP (200 nM) was incubated with the template strand of elongation scaffold DNA[13] (50 nM) for 15 minutes at RT. Next, the complex was incubated with the complementary non-template strand (50 nM) for 15 min at RT. Finally, the complex was incubated with 1 µM WT NusG, N65H NusG, R124L NusG, or N125S NusG for 10 min at RT. All complexes were assembled in the following transcription buffer: 20 mM Tris, 25 mM KGlu, 10 mM MgOAc, 1 mM DTT, 5 µg/mL BSA. Samples were immediately loaded and run on a native PAGE (4.5% acrylamide:bis solution 37.5:1, 4% glycerol, 1X TBE) for 1 hr at 15 mA. The gel was run at 4 °C. The gel was first stained with GelRed (Biotium) followed by Coomassie blue for visualization of DNA and protein respectively.

METHODS REFERENCES

61. von Hippel, P. H. & Yager, T. D. Transcript elongation and termination are competitive kinetic processes. *Proc. Natl. Acad. Sci. U. S. A.* **88**, 2307–2311 (1991).
62. von Hippel, P. H. & Yager, T. D. The elongation-termination decision in transcription. *Science* **255**, 809–812 (1992).
63. DeJesus, M. A. *et al.* Comprehensive Essentiality Analysis of the Mycobacterium tuberculosis Genome via Saturating Transposon Mutagenesis. *mBio* **8**, e02133-16 (2017).
64. Woong Park, S. *et al.* Evaluating the sensitivity of Mycobacterium tuberculosis to biotin deprivation using regulated gene expression. *PLoS Pathog.* **7**, e1002264 (2011).
65. Peek, J. *et al.* Rifamycin congeners kanglemycins are active against rifampicin-resistant bacteria via a distinct mechanism. *Nat. Commun.* **9**, 4147 (2018).
66. Boyaci, H., Chen, J., Jansen, R., Darst, S. A. & Campbell, E. A. Structures of an RNA polymerase promoter melting intermediate elucidate DNA unwinding. *Nature* **565**, 382–385 (2019).
67. Wong, A. I. & Rock, J. M. CRISPR Interference (CRISPRi) for Targeted Gene Silencing in Mycobacteria. *Methods Mol. Biol. Clifton NJ* **2314**, 343–364 (2021).
68. Murphy, K. C., Papavinasasundaram, K. & Sassetti, C. M. Mycobacterial recombineering. *Methods Mol. Biol. Clifton NJ* **1285**, 177–199 (2015).
69. Larsen, M. H., Biermann, K., Tandberg, S., Hsu, T. & Jacobs, W. R. Genetic Manipulation of Mycobacterium tuberculosis. *Curr. Protoc. Microbiol.* **Chapter 10**, Unit 10A.2 (2007).
70. Kanehisa, M. & Goto, S. KEGG: kyoto encyclopedia of genes and genomes. *Nucleic Acids Res.* **28**, 27–30 (2000).
71. Kanehisa, M. Toward understanding the origin and evolution of cellular organisms. *Protein Sci. Publ. Protein Soc.* **28**, 1947–1951 (2019).
72. Kanehisa, M., Furumichi, M., Sato, Y., Kawashima, M. & Ishiguro-Watanabe, M. KEGG for taxonomy-based analysis of pathways and genomes. *Nucleic Acids Res.* **51**, D587–D592 (2023).
73. Hicks, N. D. *et al.* Clinically prevalent mutations in Mycobacterium tuberculosis alter propionate metabolism and mediate multidrug tolerance. *Nat. Microbiol.* **3**, 1032–1042 (2018).
74. Ma, P. *et al.* Compensatory effects of M. tuberculosis rpoB mutations outside the rifampicin resistance-determining region. *Emerg. Microbes Infect.* **10**, 743–752 (2021).
75. Loiseau, C. *et al.* The relative transmission fitness of multidrug-resistant Mycobacterium tuberculosis in a drug resistance hotspot. *Nat. Commun.* **14**, 1988 (2023).
76. Hubin, E. A. *et al.* Structure and function of the mycobacterial transcription initiation complex with the essential regulator RbpA. *eLife* **6**, e22520 (2017).
77. Boyaci, H. *et al.* Fidaxomicin jams Mycobacterium tuberculosis RNA polymerase motions needed for initiation via RbpA contacts. *eLife* **7**, e34823 (2018).
