# Supplementary material for: Compensatory evolution in NusG improves fitness of drug-resistant M. tuberculosis
Source: Nature. 2024 Mar 20;628(8006):186–94. doi: 10.1038/s41586-024-07206-5 (PMC10990936; doi:10.1038/s41586-024-07206-5)

Supplementary Figures

Supplemental Figure 1: CRISPRi screens in Δ*bioA* Mtb are well correlated and capture differences in the fitness cost of the weakest possible sgRNAs

(A) Growth of 10-fold serial dilutions of Δ*bioA* RifS and βS450L Mtb on 7H10 agar plates. Note that 7H10 media is supplemented with biotin (0.5 mg/L; ~2 μM), thereby allowing growth of the Δ*bioA* Mtb auxotroph.

(B-E) Correlation heatmap of the triplicate screens depicted in Figure 1B. Panels (B) and (C) depict the Pearson correlation between sgRNAs targeting genes predicted to be essential by TnSeq[63] in the –ATc (B) or +ATc (C) cultures of the RifS library. Panels (D) and (E) depict the Pearson correlation between sgRNAs targeting genes predicted to be essential by TnSeq in the –ATc (D) or +ATc (E) cultures of the βS450L library. G = generation.

(F) Expression-fitness relationships for an example gene (*secE1*) which does not display a dose-response between sgRNA strength and fitness cost. Light grey lines (RifS) and light purple lines (βS450L) represent the fits determined by 1,000 samples from the posterior distributions. The dark lines represent the mean posterior fit. Significance was determined by comparing differences in the 95% credible regions in vulnerability, as discussed in the Methods.

(G) Cartoon depicting the F_{min} (Fitness minimum) model. F_{min} estimates the fitness cost imposed by the weakest possible sgRNAs, i.e. the expression-fitness curve at a predicted sgRNA strength of 0. See the Materials and Methods for details.

(H) Histograms showing the distributions of differences in the fitness cost imposed by the weakest possible sgRNAs (F_{min}) against *secE1* RifS (black) and βS450L (purple) libraries.

Supplemental Figure 2: NusG mutants bind RNAP similarly to WT and are rarely identified in RifR strains with known compensatory mutations

(A) The number of nonsynonymous (NS) mutations in *nusG* and known compensatory mutations in *rpoAC* in 3,252 clinical Mtb isolates from Peru (see **Figure 3A, B**).

(B) The spectrum of RifR *rpoB* mutants in compensated vs total RifR Mtb. Barplot showing the frequency of *rpoB* mutations in all clinical RifR Mtb strains ("All RifR"), strains harboring putative or confirmed compensatory mutations in *nusG* or the β protrusion ("*nusG* & β protrusion", *P-value* (two-sided) = 6.1E-17), or strains harboring compensatory mutations in *rpoA* or *rpoC* ("*rpoAC*", *P-value* (two-sided) = 0.0). Bars are colored by the strain's RifR-conferring *rpoB* mutation. "Other" indicates an alternate *rpoB* allele that was observed at a frequency >0.02 in any of the three categories described above. Rare samples containing more than one RifR conferring *rpoB* mutation (which may represent mixed infection) were removed from the frequency calculation. Statistical significance was calculated with a chi-squared test; *** p≤ 0.001.

(C) *nusG* and β protrusion compensatory mutations are found across the four main Mtb lineages. Barplot showing lineages of all clinical RifR Mtb strains ("All RifR"), strains harboring putative or confirmed compensatory mutations in *nusG* or the β protrusion ("*nusG* & β protrusion"), or strains harboring compensatory mutations in *rpoA* or *rpoC* ("*rpoAC*"). Bars are colored by the strain's lineage call. Only the four most common lineages (L1-L4) are well-represented in our dataset, and thus only these lineages are shown here.

(D) Upset plot showing the overlap between known *rpoC* or *rpoA* compensatory mutations, *nusG* nonsynonymous (NS) variants, and *rpoB* β protrusion nonsynonymous variants in an inhouse dataset of clinical Mtb isolate genome sequences[41]. See **Supplemental Data 2** for more information.

(E,F) A native gel electrophoretic mobility shift assay reveals that NusG and mutants form stable complexes with

WT core RNAP (C) and βS450L core RNAP (D). The 4.5% polyacrylamide gel was visualized with Gel Red to stain the DNA and Coomassie to stain protein. NT = non-template; T = template. For gel source data, see **Supplemental Figure 4**.

Supplemental Figure 3: Translation-related collateral vulnerabilities are not pervasively under-expressed in βS450L RifR Mtb clinical strains

(A,B) Heatmap showing the log2 fold change (L2FC) in (A) RNA or (B) protein levels between paired RifS and βS450L Mtb clinical isolates from Trauner et al.[27] Genes shown include translation-related collateral vulnerabilities annotated as tRNA synthetases, ribosomal proteins, translation initiation and elongation factors, and amino acid biosynthetic genes that were also detected both by RNAseq and quantitative proteomics in Trauner et al.[27] N0052, N0072, N0145, N0157, and N0155 denote five different paired RifS and βS450L Mtb clinical isolates. Stars denote significant differential expression between RifS and βS450L ($|L2FC| > 1$, $p\text{-adj} < 0.01$) in Trauner et al.[27]

(C) Expression-fitness relationship for *rho*. Light grey lines (RifS) and light purple lines (βS450L) represent the fits determined by 1,000 samples from the posterior distributions. The dark lines represent the mean posterior fit. Significance was determined by comparing differences in the 95% credible regions in vulnerability, as discussed in the Methods.

(D) Histogram displaying the distribution of *rho* F_{min} values in RifS (grey) and βS450L (purple) Mtb. Dashed lines represent the 95% credible regions.

Supplemental Figure 4: Source gels

(A,B) Source gels for (A) RifS and (B) RifR RNAP native gel electrophoretic mobility shift assays shown in **Supplemental Figure 2**.

Supplemental Figure 1

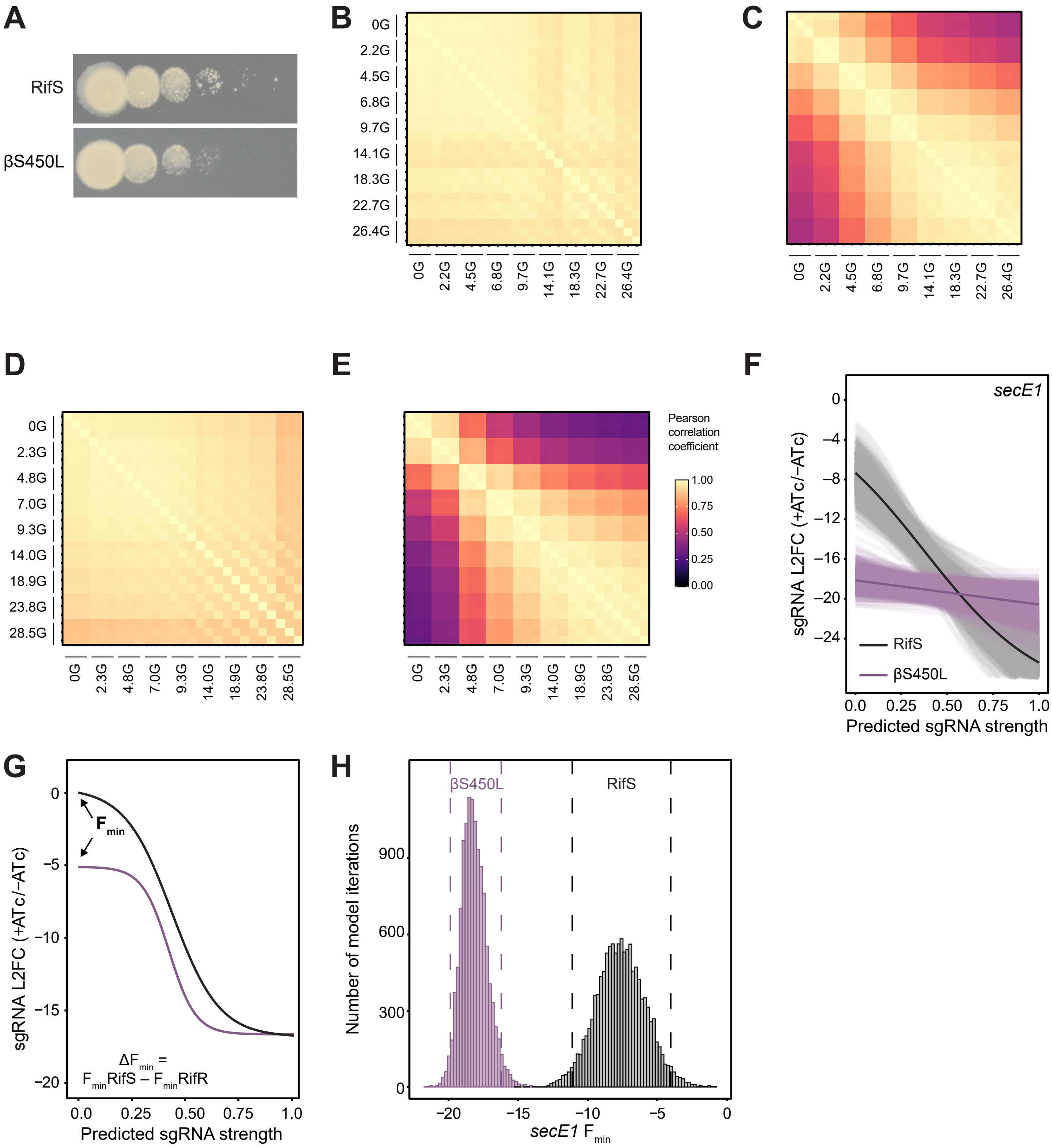

Supplemental Figure 2

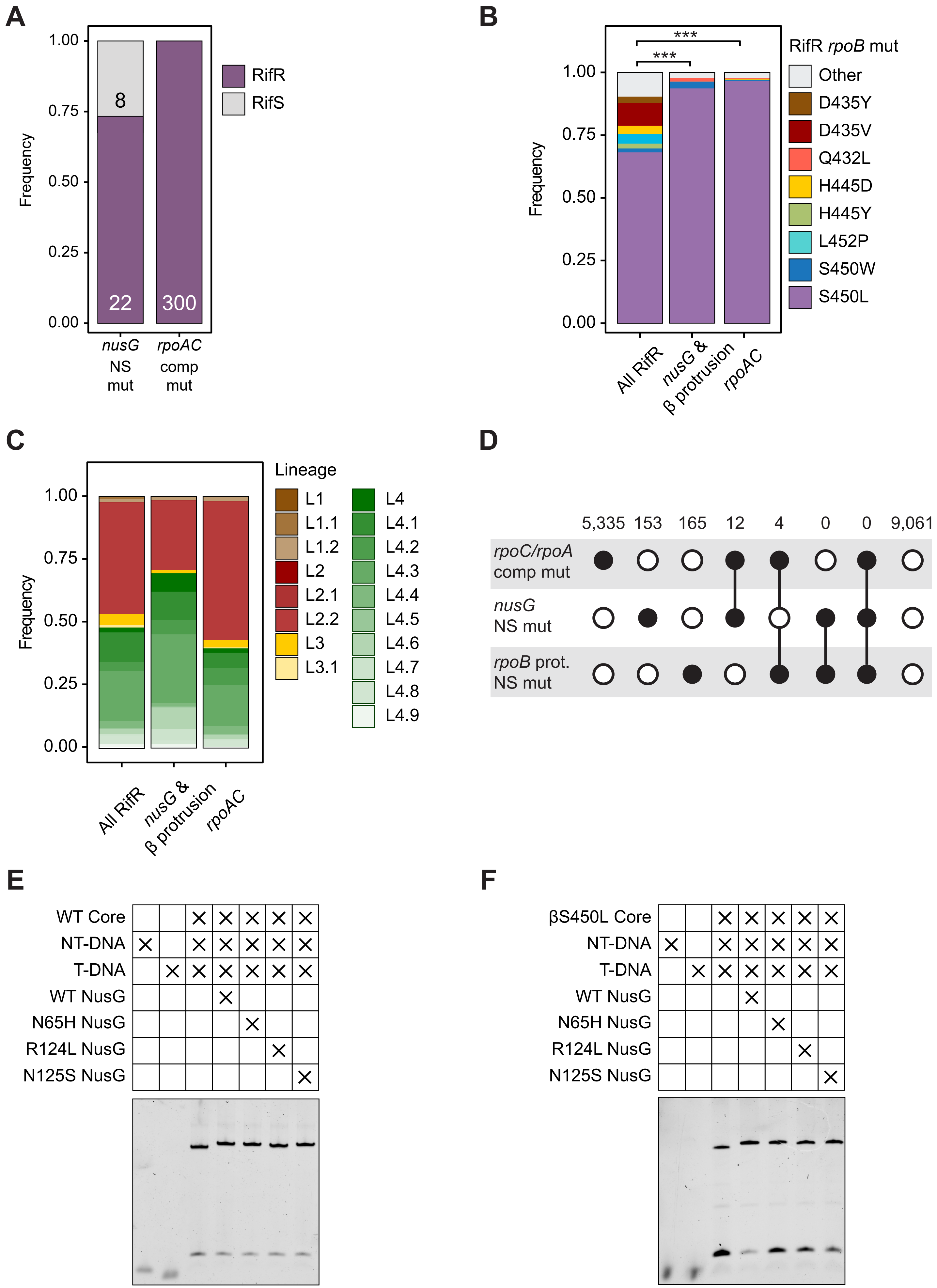

Supplemental Figure 3

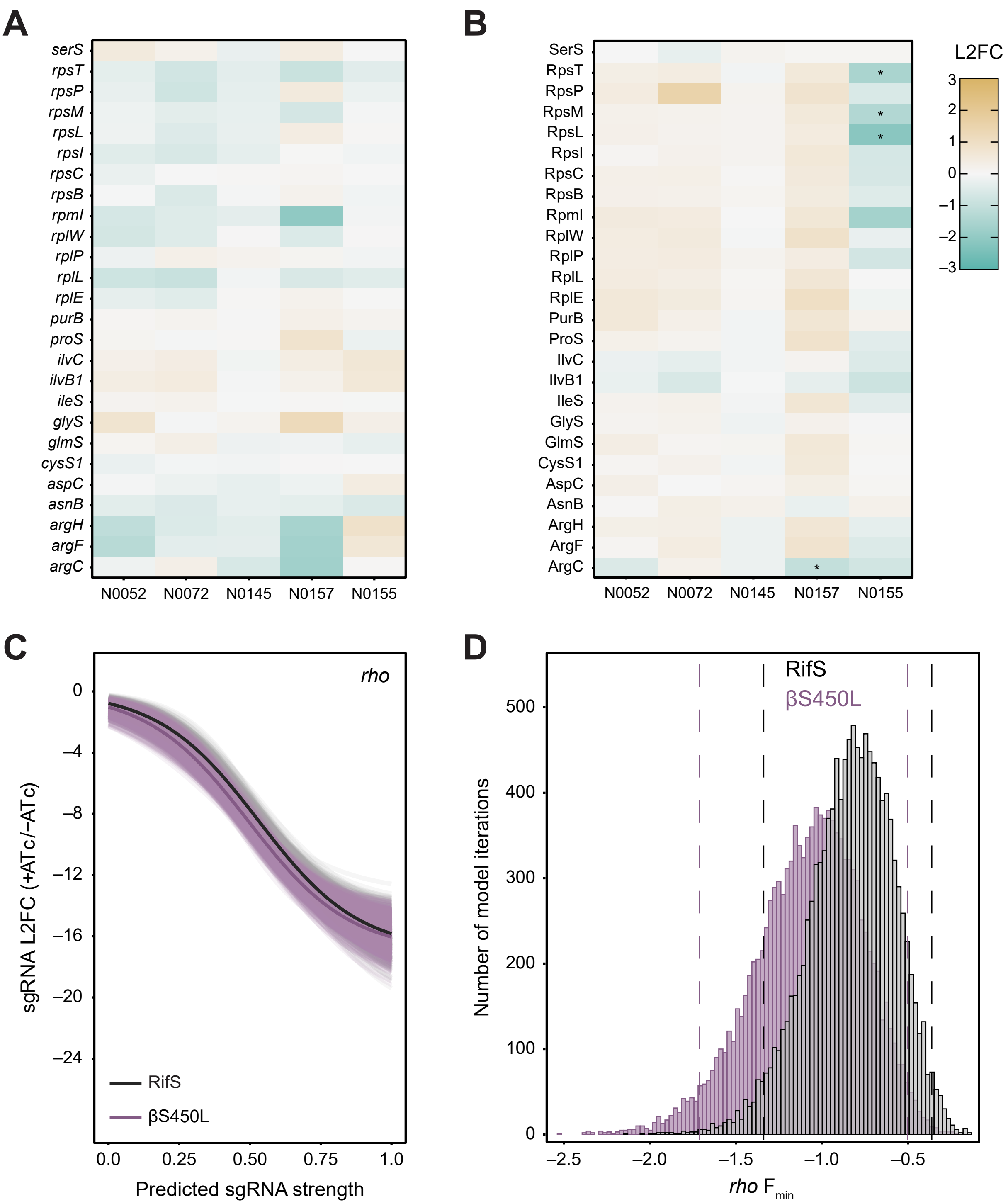

Supplemental Figure 4

A WT RNAP

WT Core			X	X	X	X	X
NT-DNA	X		X	X	X	X	X
T-DNA		X	X	X	X	X	X
WT NusG				X			
N65H NusG					X		
R124L NusG						X	
N125S NusG							X

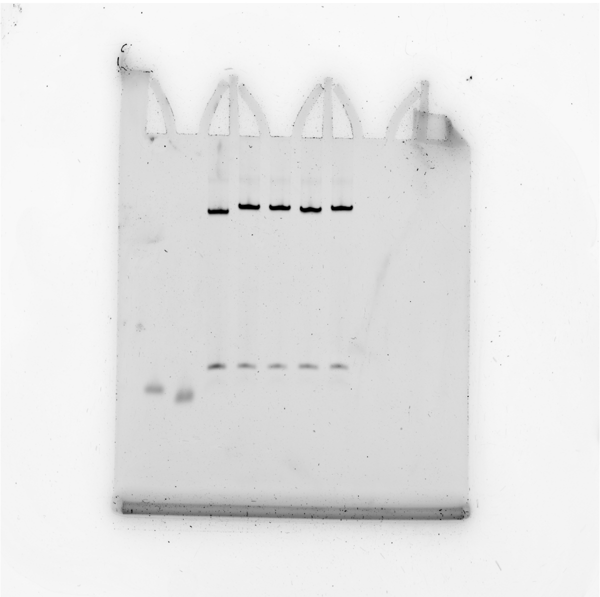

B βS450L RNAP

βS450L Core			X	X	X	X	X
NT-DNA	X		X	X	X	X	X
T-DNA		X	X	X	X	X	X
WT NusG				X			
N65H NusG					X		
R124L NusG						X	
N125S NusG							X

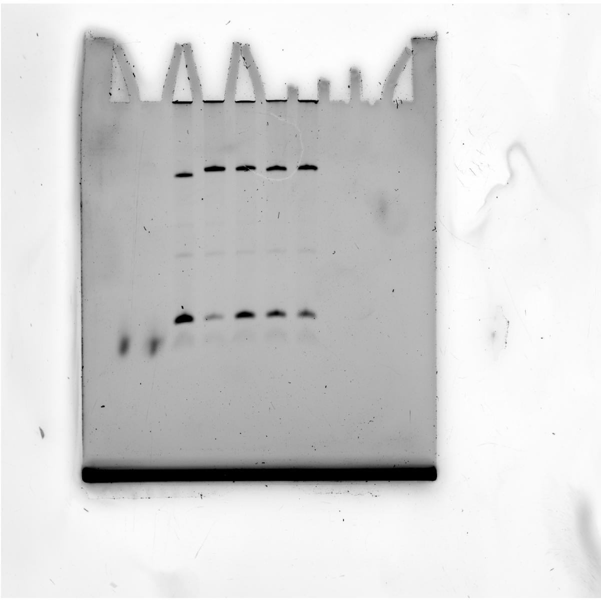

Supplement: Supplementary file 3 — Supplementary Figs. 1–4. [file 41586_2024_7206_MOESM3_ESM.pdf]
